# CaGdt1 plays a compensatory role for the calcium pump CaPmr1 in the regulation of calcium signaling and cell wall integrity signaling in *Candida albicans*

**DOI:** 10.1186/s12964-018-0246-x

**Published:** 2018-06-28

**Authors:** Linghuo Jiang, Junjun Wang, Faiza Asghar, Nathan Snyder, Kyle W. Cunningham

**Affiliations:** 1 Laboratory for Yeast Molecular and Cell Biology, School of Agricultural Engineering and Food Science, Shandong University of Technology, Zibo, Shandong China; 2 Department of Food Engineering, Weihai Ocean Vocational College, Weihai, Shandong China; 3 Department of Biology, the Johns Hopkins University, Baltimore, MD USA

**Keywords:** *Candida albicans*, Gdt1, Pmr1, Calcium homeostasis, Calcium signaling, Cell wall integrity signaling, Transcription profiling, Chs2, Chs3

## Abstract

**Background:**

*Saccharomyces cerevisiae* ScGdt1 and mammalian TMEM165 are two members of the UPF0016 membrane protein family that is likely to form a new group of Ca^2+^/H^+^ antiporter and/or a Mn^2+^ transporter in the Golgi apparatus. We have previously shown that *Candida albicans CaGDT1* is a functional ortholog of *ScGDT1* in the response of *S. cerevisiae* to calcium stress. However, how CaGdt1 together with the Golgi calcium pump CaPmr1 regulate calcium homeostasis and cell wall integrity in this fungal pathogen remains unknown.

**Methods:**

Chemical sensitivity was tested by dilution assay. Cell survival was examined by measuring colony-forming units and staining with Annexin V-FITC and propidium iodide. Calcium signaling was examined by expression of downstream target gene *CaUTR2*, while cell wall integrity signaling was revealed by detection of phosphorylated Mkc1 and Cek1. Subcellular localization of CaGdt1 was examined through direct and indirect immunofluorescent approaches. Transcriptomic analysis was carried out with RNA sequencing.

**Results:**

This study shows that *Candida albicans CaGDT1* is also a functional ortholog of *ScGDT1* in the response of *S. cerevisiae* to cell wall stress. CaGdt1 is localized in the Golgi apparatus but at distinct sites from CaPmr1 in *C. albicans*. Loss of *CaGDT1* increases the sensitivity of cell lacking *CaPMR1* to cell wall and ER stresses. Deletion of *CaGDT1* and/or *CaPMR1* increases calcium uptake and activates the calcium/calcineurin signaling. Transcriptomic profiling reveals that core functions shared by CaGdt1 and CaPmr1 are involved in the regulation of cellular transport of metal ions and amino acids. However, CaGdt1 has distinct functions from CaPmr1. Chitin synthase gene *CHS2* is up regulated in all three mutants, while *CHS3* is only up regulated in the *pmr1/pmr1* and the *gdt1/gdt1 pmr1/pmr1* mutants. Five genes (*DIE2*, *STT3*, *OST3*, *PMT1* and *PMT4*) of glycosylation pathway and one gene (*SWI4*) of the cell wall integrity (CWI) pathway are upregulated due to deletion of *CaGDT1* and/or *CaPMR1*. Consistently, deletion of either *CaPMR1* or *CaGDT1* activates the CaCek1-mediated CWI signaling in a cell wall stress-independent fashion. Calcineurin function is required for the integrity of the cell wall and vacuolar compartments of cells lacking both *GDT1* and *CaPMR1*.

**Conclusions:**

CaPmr1 is the major player in the regulation of calcium homeostasis and cell wall stress, while CaGdt1 plays a compensatory role for CaPmr1 in the Golgi compartment in *C. albicans*.

**Electronic supplementary material:**

The online version of this article (10.1186/s12964-018-0246-x) contains supplementary material, which is available to authorized users.

## Plain English summary

Congenital disorders of glycosylation (CDG) are inherited diseases with most of their genetic defects affecting the glycosylation process. The human ortholog for yeast ScGdt1, TMEM165, is one of these CDG-associated Golgi proteins and might function as a Ca^2+^/H^+^ antiporter and/or a Mn^2+^ transporter in the Golgi. This study provides evidence that the functional ortholog of ScGdt1, *Candida albicans* CaGdt1 plays a compensatory role for CaPmr1, the Ca^2+^/Mn^2+^ ATPase that is required for Ca^2+^ and Mn^2+^ transport into the Golgi and involved in Ca^2+^ dependent protein sorting processing. CaGdt1 and CaPmr1 work together at distinct sites of the Golgi apparatus to regulate the response of this human fungal pathogen to cell wall and ER stresses. Calcineurin function is required for the survival of *C. albicans* cells lacking both CaGdt1 and CaPmr1. These findings would contribute to our understanding of molecular mechanisms regulating TMEM165-associated CDG.

## Background

Calcium ions regulate growth and programmed cell death as well as muscle contraction in the heart and taste in the mouth [1]. Calcium/calcineurin signaling pathway is highly conserved in eukaryotic cells. Functional counterparts of yeast calcium channels, pumps and exchangers exist and function in similar fashions in mammalian cells [2]. In *Saccharomyces cerevisiae*, calcium homeostasis is regulated through calcium transporters and sequestrates in the plasma and organelle membranes [2–4]. The plasma membrane calcium channel allows Ca^2+^ influx in response to endoplasmic reticulum (ER) stress and pheromones, while the vacuolar Yvc1 releases Ca^2+^ into the cytosol in response to hypotonic shock [4, 5]. Excess calcium ions are transported into the vacuole for storage through the vacuolar calcium pump Pmc1 and the Ca^2+^/H^+^ exchanger Vcx1; or into the ER/Golgi secretory pathway via the calcium pump Pmr1 and the Ca^2+^/H^+^ exchanger Gdt1 [3, 4, 6]. Expression of *PMR1* is positively controlled by the calcium/calcineurin signaling pathway and negatively controlled by the Rim101/Nrg1 pathway in *S. cerevisiae* [7]. Functional counterparts of Ca^2+^ transporters and channels have been characterized in *Candida albicans* [8–11]. Rch1 is a novel negative regulator of calcium uptake in the plasma membrane of *S. cerevisiae* and *C. albicans* [12–15].

*C. albicans* is the most common human fungal pathogen in immunocompromised patients [16, 17]. In *C. albicans*, there are four MAP kinases, Mkc1, Cek1, Cek2 and Hog1, with the former two mediating the cell wall integrity (CWI) pathway [18–21]. The cell wall of *C. albicans* is important in the interaction with its host during infection [16, 22]. Deletion of *CaPMR1* causes *C. albicans* cells to be hypersensitive to cell wall stress and to constitutively activate the Mkc1-mediated CWI signaling, which leads to a defect in glycosylation of cell wall proteins and thereby a weakened cell wall and reduced virulence [10]. *C. albicans* cells lacking *MKC1* or *CEK1* also show a reduced virulence in the mouse model of systemic infection [23, 24]. Therefore, properly regulated CWI signaling is required for the virulence of *C. albicans*.

*S. cerevisiae* ScGdt1 and its human transmembrane protein 165 (TMEM165) belong to a well-conserved family of membrane proteins named UPF0016 (Uncharacterized Protein Family0016; Pfam PF01169), which exist in 919 bacterial species and 409 eukaryotic species [25]. Mutations of TMEM165 are linked to a subtype of inborn metabolic diseases affecting the glycosylation pathway, and TMEM165 is a functional homolog of the Golgi-localized ScGdt1 [7]. ScGdt1 may act as a Ca^2+^/H^+^ antiporter and plays a major role in the calcium response induced by osmotic shock in the absence of ScPmr1 [26, 27]. Here, we have characterized CaGdt1, the *C. albicans* homolog of *S. cerevisiae* ScGdt1, in the response to calcium and cell wall stress. CaGdt1 and CaPmr1 localize to distinct sites in the Golgi apartment. Transcriptomic profiling reveals overlapping and distinctive functions between CaGdt1 and CaPmr1. In addition, we demonstrate that CaGdt1 is involved in the Cek1-mediated, but not the Mkc1-mediated, cell signaling.

## Methods

### Strains and reagents

*C. albicans* and *S. cerevisiae* strains, plasmids and primers are listed in Tables 1 and 2, and Additional file 1: Table S1, respectively. All strains were routinely grown at 30 °C in YPD medium or SD medium (0.67% yeast nitrogen base without amino acids, 2% glucose, and auxotrophic amino acids as needed). Chemical reagents were obtained from Sigma.

**Table 1:** Strains used in this study

Strain	Genotype	Source
*S. cerevisiae*		
BY4741	*MATa his3Δ1 leu2Δ0 met15Δ0 ura3Δ0*	Invitrogen Inc.
WJSC11	BY4741 *gdt1::kanMX4*	Invitrogen Inc.
WJSC12	BY4741 *pmr1::kanMX4*	Invitrogen Inc.
WJSC13	BY4741 *gdt1::natR pmr1::kanMX4*	Invitrogen Inc.
*C. albicans*		
RM1000	*ura3Δ::λimm434/ura3Δ::λimm434 his1::hisG/his1::hisG*	[13]
WJCA110	RM1000 *PMR1/PMR1::HA-HIS1*	This study
WJCA111	RM1000 *PMR1/PMR1:: HA-HIS1 GDT1/GDT1:: GFP-URA3*	This study
CAI4	*ura3::λimm434/ura3::λimm434*	[10]
NGY98	CAI4 *pmr1::hisG/pmr1::hisG*	[10]
WJCAG22	CAI4 *gdt1::hisG/gdt1::FRT*	[30]
WJCAG25	CAI4 *pmr1::hisG/pmr1::hisG gdt1::hisG/gdt1::FRT*	This study
WJCAG26	CAI4 *RPS1/rps1::*CIp10	This study
WJCAG27	CAI4 *pmr1::hisG/pmr1::hisG RPS1/rps1::*CIp10	This study
WJCAG28	CAI4 *gdt1::hisG/gdt1:: FRT RPS1/rps1::*CIp10	This study
WJCAG29	CAI4 *pmr1::hisG/pmr1::hisG gdt1::hisG/gdt1:: FRT RPS1/rps1::*CIp10	This study
WJCA100	CAI4 *pmr1::hisG/pmr1::hisG gdt1::FRT/GDT1*	This study
WJCA102	CAI4 *pmr1::hisG/pmr1::hisG gdt1::FRT/GDT1:: GFP-URA3*	This study
WJCA201	CAI4 *MKC1/MKC1:: HA-URA3*	This study
WJCA202	CAI4 *gdt1::hisG/gdt1:: FRT MKC1/MKC1::HA-URA3*	This study
WJCA203	CAI4 *pmr1::hisG/pmr1::hisG MKC1/MKC1::HA-URA3*	This study
WJCA204	CAI4 *pmr1::hisG/pmr1::hisG gdt1::hisG/gdt1:: FRT MKC1/MKC1::HA-URA3*	This study
WJCA205	CAI4 *CEK1/CEK1::HA-URA3*	This study
WJCA206	CAI4 *gdt1::hisG/gdt1:: FRT CEK1/CEK1::HA-URA3*	This study
WJCA207	CAI4 *pmr1::hisG/pmr1::hisG CEK1/CEK1::HA-URA3*	This study
WJCA208	CAI4 *pmr1::hisG/pmr1::hisG gdt1::hisG/gdt1:: FRT CEK1/CEK1::HA-URA3*	This study
WJCA209	CAI4 (UTR2p::*Lac*Z reporter)	This study
WJCA210	CAI4 *gdt1::hisG/gdt1:: FRT* (UTR2p::*Lac*Z reporter)	This study
WJCA211	CAI4 *pmr1::hisG/pmr1::hisG* (UTR2p::*Lac*Z reporter)	This study
WJCA212	CAI4 *pmr1::hisG/pmr1::hisG gdt1::hisG/gdt1:: FRT* (UTR2p::Lac Z reporter)	This study

**Table 2:** Plasmids used in this study

Name	Description	Source
pCR4	*C. albicans* expression vector with *CaURA3* marker	[13]
p5921	*C. albicans URA3* blaster cassette	[29]
pCR4-CaGDT1	Full-length *CaGDT1* gene in pCR4	This study
pHAC181	*S. cerevisiae* 2μ expression vector with *LEU2* marker	[31]
pHAC181-CaGDT1	Full-length *CaGDT1* gene in pHAC181	This study
pGFP-URA3	GFP integration vector with *URA3* marker	[34]
pFA-HA-URA3	HA-epitope integration vector with *CaURA3* marker	[34]
pFA-HA-HIS1	HA-epitope integration vector with *CdHIS1* marker	[34]
pSFS2	the SAT1 flipper cassette	[28]
pKC1	Two flanking regions of *CaGDT1* at either side of the *hisG-URA3-hisG* cassette in p5921	This study
pGP8	Plasmid containing the 1-kb promoter of *UTR2* fused to the *lac*Z reporter	[13]

### Construction of *C. albicans* mutants

We constructed the double-gene deletion mutant by replacing two *CaGDT1* alleles in the *pmr1/pmr1* mutant in the CAI4 background [10], with the *SAT1* flipper cassette [28] and the *his*G-*URA3*-*his*G cassette [29], respectively, as described previously [30]. Genotypes of the resultant mutants were confirmed by PCR (data not shown).

### DNA manipulation

The *2*150-bp *CaGDT1* gene containing its promoter, ORF and 3′ terminator was amplified from genomic DNA with primers GDT1 (BamHl) –UP and GDT1 (BamHl) –DOWN, and cloned into the *C. albicans* expression vector pCR4 [13], which yielded pCR4-CaGDT1. Similarly, we amplified the *CaGDT1* gene using the primers GDT1 (Kpnl) –UP and GDT1 (Sphl) –DOWN, and cloned it into the *Kpn*l and *Sph*l sites of *S. cerevisiae* vector pHAC181 [31], which yielded pHAC181-CaGDT1.

### Growth test

*S*trains were cultured overnight in liquid YPD or SD-URA medium at 30 °C. Overnight cultures were serially diluted by 10-fold, and spotted onto appropriate plates. Phenotypes were recorded after plates were incubated for 2–3 days at 30 °C.

### ^45^Ca^2+^ uptake assays

Cellular accumulation of Ca^2+^ was measured basically as described [32]. Cells were inoculated in YPD medium containing 5 μCi ml^− 1 45^CaCl_2_ with a specific activity of 9.6 × 10^6^ cpm ml^− 1^ (PerkinElmer), and grown to log phase at 30 °C in 96-well filtration plates. Cultures were harvested by filtration, washed four times with ice-cold buffer (5 mM Na-HEPES, pH 6.5, 10 mM CaCl_2_, 150 mM NaCl), and dried at room temperature. The filters were counted using a TopCount NXT (Packard) liquid scintillation counter.

### Assays of β-galactosidase activity

The *CaUTR2*p-*lac*Z reporter in the pGP8 plasmid [13] was integrated into the *CaUTR2* locus in the wild type CAI4, the *gdt1/gdt1* mutant, the *pmr1/pmr1* mutant and the *gdt1/gdt1pmr1/pmr1* mutant. Three independent transformants of each strain were assayed for β-galactosidase activity as described previously [13].

### Analysis of cell survival and cell integrity

Test strains were grown to log phase before the culture was divided into two parts, which were treated with 25 μg ml^− 1^ CsA and ethanol (as control), respectively. Two parts were further incubated with shaking for 1 h, 2 h, 4 h and 8 h, respectively, before samples were taken for measuring colony-forming units (clonogenic survival) as well as co-staining with 20 μg ml^− 1^ Annexin V-FITC and 50 μg ml^− 1^ propidium iodide (CLONETECH Laboratories, USA). Vacuoles in these cells were labelled in vivo with N-(3-triethylammoniumpropyl)-4-(6 [4-(diethylamino) phenyl) hexatrienyl] pyridinium dibromide (FM4–64; Molecular Probes, USA) as described [33].

### Protein extract and western blot analysis

Cells were grown to mid-exponential phase, and treated with 50 μg ml^− 1^ Calcofluor white for 2 h before they were collected for protein extraction and Western blot analysis as described [7]. The anti-phospho-p44/42 MAPK (Thr^202^ /Tyr^204^) antibody (Cell signaling, USA) was used to simultaneously detect phosphorylated forms of both CaMkc1 and CaCek1 [23, 24]. Anti-HA antibody (Abmart, Shanghai) was used to detect CaMkc1-HA and CaCek1-HA proteins [7].

### Chromosomal integration of green fluorescent protein (GFP) and HA epitope

GFP tagging cassette was PCR-amplified with CaGDT1-GFP-UP and CaGDT1-GFP-DOWN from the pGFP-URA plasmid [34], using primers CaGDT1-GFP-UP and CaGDT1-GFP-down, and integrated into the heterozygous mutant (WJCA102). Genotype of the CaGDT1-GFP tagged strain with correct integration was confirmed by PCR (Additional file 1: Figure S2B). To examine the co-localization between CaGdt1 and CaPmr1, we first chromosomally tagged HA epitope to the C-terminus of CaPMR1 in the wild-type RM1000 strain, yielding RM1000 PMR1/PMR1::HA-HIS1 strain (WJCA110). The HA-HIS1 cassette was PCR-amplified with primers PMR1-HA-HIS-UP and PMR1-HA-HIS-DOWN from the pFA-HA-HIS1 plasmid [34, 35]. We subsequently tagged the GFP to the C-terminus of CaGDT1 in the WJCA110 strain, which generated the RM1000 PMR1/PMR1::HA-HIS1 GDT1/GDT1:: GFP-URA3 strain (WJCA111). Genotypes of WJCA110 and WJCA111 strains were confirmed by PCR (Additional file 1: Figure S2B).

Two HA-URA3 cassettes were PCR-amplified with primer pairs, MKC1-HA-UP/ MKC1-HA-DOWN and CEK1-HA-UP and CEK1-HA-DOWN, respectively, from the pFA-HA-URA3 plasmid [34], to chromosomally tag the HA epitope to the C-termini of CaMKC1 and CaCEK1 in relevant strains. Their genotypes were confirmed by PCR (Additional file 1: Figure S2C and S2D).

### Co-localization of GDT1-GFP and PMR1-HA proteins

Cells of the WJCA111 strain was grown to log-phase in SD-URA-HIS medium before they were treated with 100 mM CaCl_2_ or 1 mM EGTA for 2 and 6 h, respectively. Cells were treated with 3.7% formaldehyde for 10 min and incubated in fixation buffer (0.1 M KH2PO4,0.5 M MgCl2, 3.7% formaldehyde, pH 6.5) for 20 min. Cells were then resuspended in 1 ml buffer (0.1 M KH_2_P_4_, 1.2 M sorbitol, pH 6.5) supplemented with 4 μl of beta-mercaptoethanol and 4 μl of 5 mg ml^− 1^ Zymolyase 20 T (Seikagaku Biobusiness), and incubated at 37 °C for 20 min. Spheroplasts were washed twice with 1 ml of PBS buffer, and suspended in 100 μl of PBS supplemented with 0.05% Tween 20 (PBST).

BSA of 400 μg ml^− 1^ was added to the spheroplast mixture, and incubated for 20 min before mouse anti-HA antibodies was added at a dilution of 1:500. The mixture was incubated for 2 h, and washed twice with PBST before goat anti-mouse IgG conjugated to Alexa Fluor 555 (Invitrogen, USA) was added. The mixture was incubated in dark for 45 min before spheroplasts were collected, washed three times, and visualized under a Nikon 80i microscope equipped with DS-U2 CCD. The images were acquired at 1000 × and processed using the NIS – ELEMENTS F3.0 software.

### Co-localization of GDT1-GFP protein and Golgi-tracker red dye

For Golgi apparatus labeling, the *pmr1/pmr1 GDT1-GFP/gdt1* cells were grown to log-phase in SD-URA, harvested, washed twice with PBS, and suspended in 200 μl PBS supplemented with 4 μl beta-mercaptoethanol and 15 U lyticase enzyme (Sigma). Cells was incubated at 37 °C for 40 min to partially digest the cell wall, and were washed once with PBS and suspended in 200 μl PBS before they were mixed with 2 μl Golgi-Tracker Red dye (33.3 mg ml^− 1^) (Beyotime Institute of Biochemistry, China) and incubated at 4 °C for 30 min. Cells were then washed twice and incubated in 1 ml SD-URA at 30 °C for 30 min, before they were visualized by the Nikon 80i fluorescent microscope.

### RNA extraction and qRT-PCR assays

Total RNA was extracted as described previously (12). Contaminated genomic DNA in the reverse transcription products was examined by PCR of 35 cycles using a pair of primers CaACT1-g-UP and CaACT1-g/m-DOWN flanking the intron of the *C. albicans ACT1* gene. Real-time qPCR was performed in the CFX96™ Real-Time System I (BIO-RAD) using SYBR Premix EX Taq™ II (TAKARA Biotechnology, China). All assays were performed in duplicate. For quantification, the abundance of each gene was determined relative to the transcript of *CaACT1*.

### Transcript profiling and data analysis

Total RNA samples were extracted from the wild type CAI4, the *gdt1/gdt1*, the *pmr1/pmr1*, and the *gdt1/gdt1 pmr1/pmr1* mutants grown to log phase in SD-URA at 30 °C. RNA integrity was evaluated using an Agilent 2100 Bioanalyzer (Agilent Technologies, USA). RNA-seq libraries were constructed using Illumina’s TruSeq RNA Sample Preparation Kit (Illumina Inc., USA). RNA sequencing, data analysis and sequence assembly were performed by Beijing BioMarker Technologies (Beijing, China). Preparation of the paired-end libraries and sequencing were performed following standard Illumina methods and protocols. The mRNA-seq library was sequenced on the Illumina Hiseq 2500 and Illumina Genome Analyzer system.

To obtain high-quality clean read data for de novo assembly, raw reads from mRNA sequencing were filtered by discarding the reads with adaptor contamination, masking low-quality reads with ambiguous ‘N’ bases and removing reads in which more than 10% bases had a Q-value < 20. Clean reads were assembled into full-length transcriptome from RNA-Seq data with the reference genome (http://www.candidagenome.org/).

### Digital analysis of gene expression

Gene expression levels were measured as numbers of reads, and normalized with RPKM in RNA-Seq analyses as described [36]. Differentially expressed genes were identified in pair-wise comparison with the IDEG6 software [37], and all statistical test results were corrected for multiple testing with the Benjamini–Hochberg false discovery rate (FDR<0.01). Gene transcripts were considered to be significantly differentially expressed if the adjusted *P* value was <0.001 and there was at least a two-fold change (>1 or <-1 in log 2 ratio value) in PKM between two libraries.

### Semi-quantitation of gene expression levels by RT-PCR

To confirm the RNA-sequencing data, we carried out semi-quantitative RT-PCR with reverse transcription products that were treated to remove their contaminated genomic DNA. RT-PCR was carried out as described previously [12]. PCR products were separated on agarose gel and stained with ethidium bromide. DNA contents in all lanes of the gel were quantified and analyzed using the Bio-Rad Gel DocTM XR+ System and the Image Lab software (Version 4.0.1). Relative levels of target gene transcripts in each strain were calculated using *CaPGK1* as the internal control.

### Statistical analysis

Significant differences were analysed by GraphPad Prism version 4.00. *P* values of < 0.05 were considered to be significant.

## Results

### *CaGDT1* complements the function of *ScGDT1* in cell wall stress

As reported previously [30], expression of *CaGDT1* in the *S. cerevisiae* vector, pHAC181, fully suppressed the sensitivity of the *Scgdt1* single-gene deletion mutant to 0.6 M and 0.7 M CaCl_2_ as well as the sensitivity of the *Scgdt1/Scpmr1* double-gene deletion mutant to 0.4 M CaCl_2_ (Additional file 1: Figure S1A). In addition, expression of *CaGDT1* reverted the sensitivity level of the *Scgdt1/Scpmr1* double-gene deletion mutant to that of the *Scpmr1* single-gene deletion mutant under higher concentrations of CaCl_2_ (0.6 M and 0.7 M) (Additional file 1: Figure S1A). Therefore, *CaGDT1* is a functional homolog of ScG*DT1* in calcium stress [30]. Although *S. cerevisiae* cells lacking *ScGDT1* were not sensitive to 5 mM EGTA, 0.4 M CaCl_2_, 100 μg ml^− 1^ Congo red (CR) and 50 μg ml^− 1^ Calcofluor white (CFW) [7], we found that deletion of *ScGDT1* increased the sensitivity of cells lacking *ScPMR1* to all these reagents (Additional file 1: Figure S1A). Expression of *CaGDT1* reverted the sensitivity level of the *Scgdt1/Scpmr1* double-gene deletion mutant to that of the *Scpmr1* single-gene deletion mutant under these cell-wall stress conditions (Additional file 1: Figure S1A). Taken together, these data demonstrate that CaGdt1 complements the function of ScGdt1 in the response of *S. cerevisiae* cells to cell-wall stress.

### *CaGDT1* shows genetic interactions with *CaPMR1* in the response to cell wall and ER stress

**Fig. 1:**
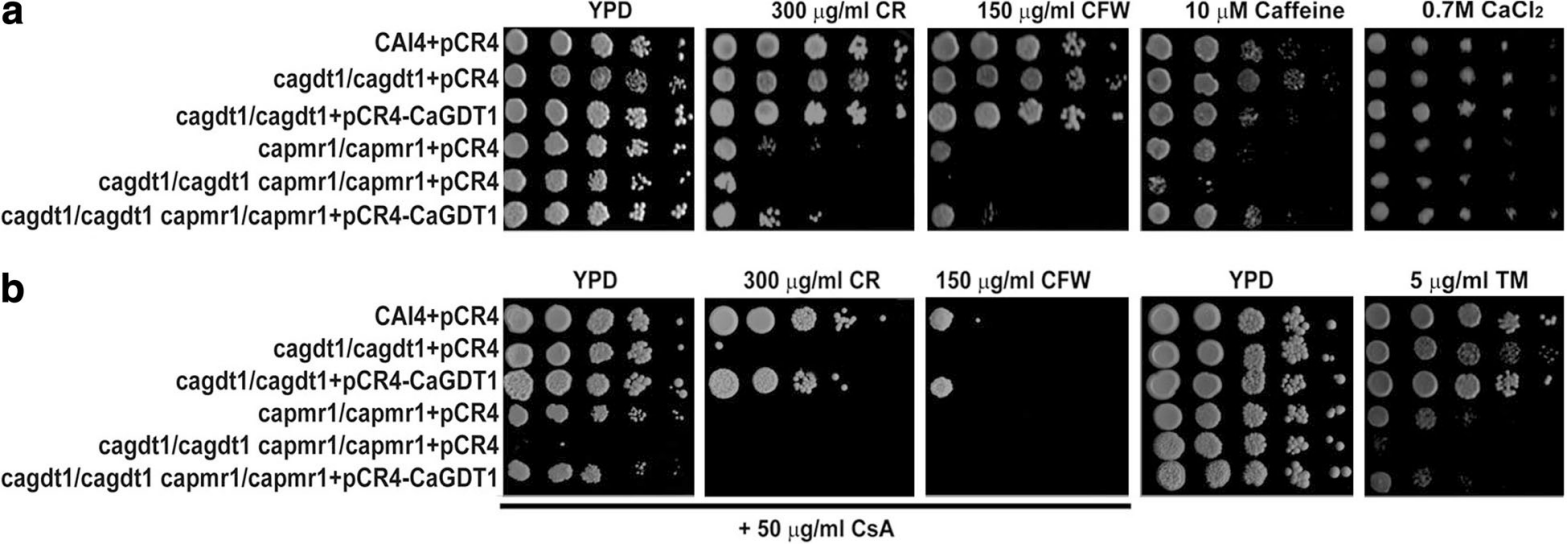
Roles of *CaGDT1* and *CaPMR1* in the sensitivity to cell wall-perturbing agents and calcium. Phenotypes of the *Candida albicans* single-gene *gdt1/gdt1* and *pmr1/pmr1* mutants as well as the double-gene *gdt1/gdt1 pmr1/pmr1* mutant in the sensitivity to cell-wall perturbing agents in the absence (**a**) or presence of cyclosporine A (CsA) (**b**). Indicated *C. albicans* strains containing the pCR4 vector or the pCR4-CaGDT1 recombinant plasmid were grown overnight in SD-URA medium, serially diluted by 10 times and spotted onto plates. Plates were incubated at 30 °C for 3 days before photos were taken. CR, Congo red. CFW, calcofluor white. TM, tunicamycin

Since mutations of human TMEM165, the CaGdt1 homolog, affect the glycosylation pathway [25, 38], we tested cells lacking *CaGDT1* against cell wall and ER stresses. Surprisingly, *C. albicans* cells lacking *CaGDT1* did not show significant alteration in their sensitivity to CR, CFW, caffeine and tunicamycin (Fig. 1a).

Unlike *ScGDT1*, *C. albicans* cells lacking *CaGDT1* were not sensitive to 0.7 M CaCl_2_ (Fig. 1a; Additional file 1: Figure S1A; [25]). However, *ScGDT1* genetically interacts with *ScPMR1* in the calcium sensitivity of *S. cerevisiae* cells [25]. We next investigated possible interactions between *CaGDT1* and *CaPMR1* in cell wall stress. As reported previously [10], cells lacking *CaPMR1* were hypersensitive to CR, CFW, caffeine and tunicamycin (Fig. 1a and b). Further deletion of *CaGDT1* increased the sensitivity of cells lacking *CaPMR1* to CR, CFW, caffeine and tunicamycin, which could be reversed by the introduction of the *CaGDT1* gene (Fig. 1a and b). These results demonstrate that *CaGDT1* has an additive effect on *CaPMR1* in the response of *C. albicans* cells to cell wall and ER stresses.

### Calcineurin function is required for *C. albicans* cells in response to cell wall stress

Further deletion of *CaGDT1* led cells lacking *CaPMR1* hypersensitive to cyclosporine A (CsA) and FK506, specific inhibitors of calcineurin, in YPD medium with or without cell wall stress (Fig. 1b; Additional file 1: Figure S1B). This indicates that normal growth of *C. albicans* cells lacking both *CaGDT1* and *CaPMR1* requires the calcineurin function.

The homozygous mutant for *CaGDT1* became hypersensitive to CR and CFW in the presence of 50 μg ml^− 1^ CsA, and its sensitive degree was comparable to that of *C. albicans* cells lacking both *CaGDT1* and *CaPMR1* on YPD plates containing these reagents without CsA (Compare Fig. 1b to a). Furthermore, addition of CsA in YPD plates made *C. albicans* cells more sensitive to CR and CFW independent of *CaGDT1* and *CaPMR1* (Compare Fig. 1b to a). This suggests that calcineurin function is additive to the overlapping function between CaGdt1 and CaPmr1 in the response of *C. albicans* cells to cell wall stress.

### Inhibition of calcineurin promotes cell death and disrupts cell wall and vacuolar integrity of cells lacking both *CaGDT1* and *CaPMR1*

**Fig. 2:**
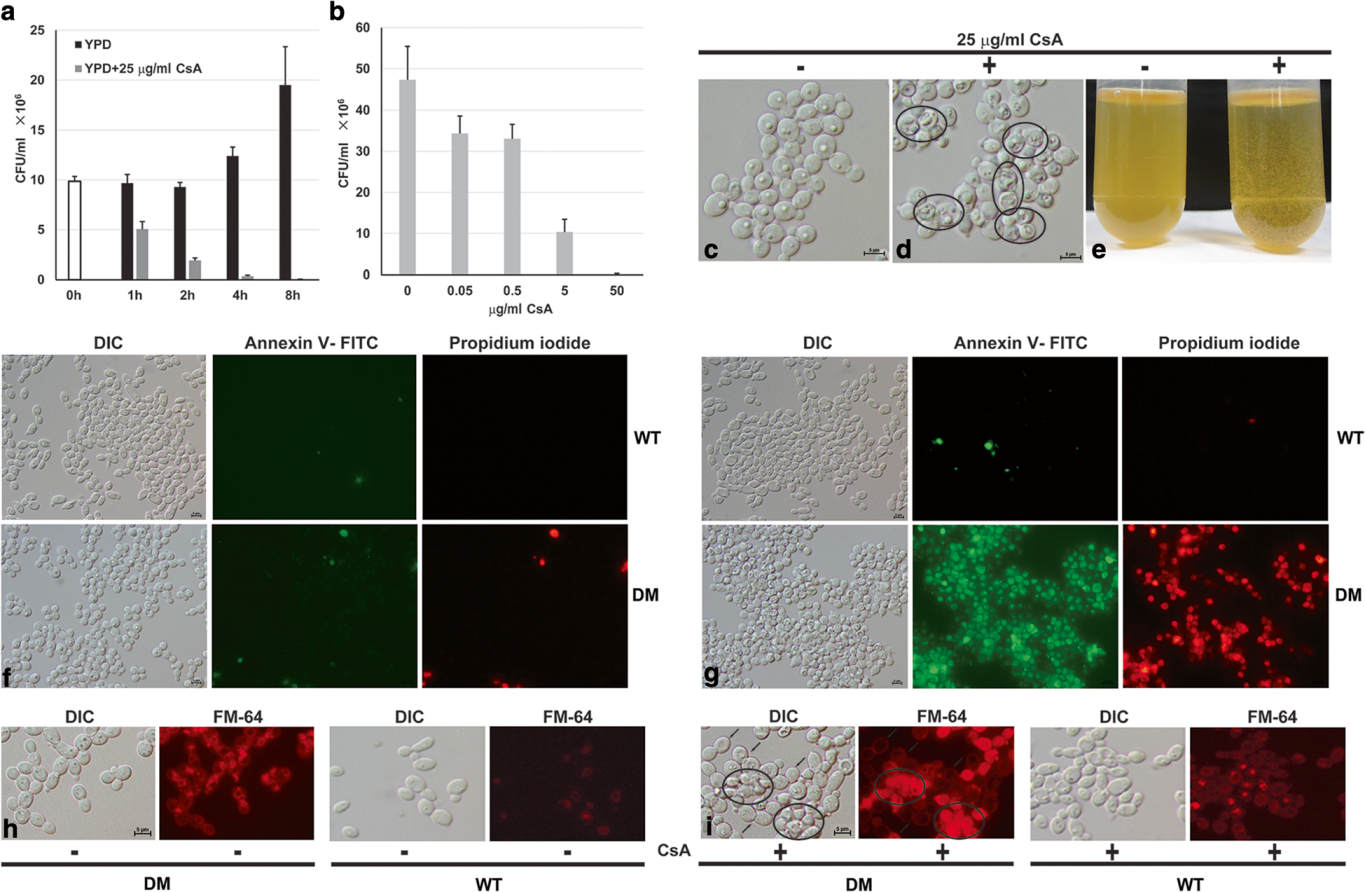
Calcineurin function is essential for cell survival and integrity of *C. albicans* cells lacking both *CaGDT1* and *CaPMR1*. Quantification of clonogenic survival of cells treated with CsA for different time (**a**) and different concentrations (**b**) as indicated. Clonogenic survival was determined by plating cells onto YPD plates, and colony-forming unit (CFU) was counted manually. Data are means of three independent experiments. Empty bar indicates the colony-forming unit (CFU) of the cell culture immediately before the CsA treatment. Black bars and grey bars represent CFUs of cell cultures not treated and treated with 25 μg ml^− 1^ CsA, respectively. Morphology of cells untreated (**c**) and treated with CsA for 8 h (**d**). **e** Cell cultures untreated (left test tube) and treated with CsA for 8 h (right test tube). Representative epifluorescent micrographs of cells untreated (**f**) and treated with CsA for 8 h (**g**) after being co-stained with both Annexin V-FITC and propidium iodide. Epifluorescent micrographs of cells untreated (**h**) and treated with CsA for 4 h (**i**), after being incubated with the vacuole indicator dye FM4–64 for 30 min. The wild-type (WT) and the double-gene *gdt1/gdt1 pmr1/pmr1* mutant (DM) were cultured at 30 °C in YPD to log phase before they were treated with CsA of 25 μg/ml for these experiments. Cells with normal cell morphology but without intracellular FM4–64 staining are indicated with arrows, while cells with abnormal internal structures are circled

To further investigate the growth defect of *C. albicans* cells lacking *CaGDT1* and *CaPMR1* in the presence of CsA, we determined their clonogenic survival ability by measuring their colony-forming units. In response to 25 μg ml^− 1^ CsA in YPD medium, cells of the *gdt1/gdt1 pmr1/pmr1* mutant lost their viability in both time-dependent and dose-dependent fashions (Fig. 2a and b). However, CsA treatment of the wild type did not affect its viability (Data not shown). This indicates that CsA promotes the death of cells lacking both *CaGDT1* and *CaPMR1*.

Compared to untreated cells (Fig. 2c), most of cells treated with 25 μg ml^− 1^ CsA for 8 h were abnormal in their internal structures (Fig. 2d), and appeared to be flakes in culture solution (Fig. 2e). To examine their cell wall integrity, we co-stained these cells with Annexin V-FITC (green fluorescence) and propidium iodide (red fluorescence), which measure externalization of phosphatidylserine and loss of membrane integrity of yeast cells, two indicators of cell death [39, 40]. Only a few of the wild-type and the mutant cells untreated with CsA were stained (Fig. 2f). Comparing to the CsA-treated wild-type with only a few of stained cells, most of mutant cells treated with CsA were stained, by these two dyes (Fig. 2g), indicating susceptible cell walls of CsA-treated mutant cells for an easy entry of these dyes.

To understand the nature of the abnormal intracellular compartments in CsA-treated cells lacking both *CaGDT1* and *CaPMR1* (Fig. 2d), we examined the integrity of their vacuoles with the dye FM4–64 [33]. Untreated wild-type and mutant cells showed a uniform staining pattern of vacuoles, indicating normal endocytosis and enrichment of FM4–64 in the vacuolar membrane (Fig. 2h). Wild-type cells treated with CsA showed a uniform staining pattern, while mutant cells treated with CsA exhibited various staining patterns (Fig. 2i), including cells with normal intracellular compartments and no intracellular staining, indicating their lost ability of transporting FM4–64 through endocytosis, and cells with abnormal intracellular compartments and strong whole-cell staining, indicating their disrupted vacuolar structures. Taken together, these results suggest that calcineurin function is essential for the cell survival as well as the cell wall and vacuolar integrity of cells lacking both *CaGDT1* and *CaPMR1*.

### Calcium uptake and activation of the calcium/calcineurin signaling in *C. albicans* cells lacking *CaGDT1* and *CaPMR1*

**Fig. 3:**
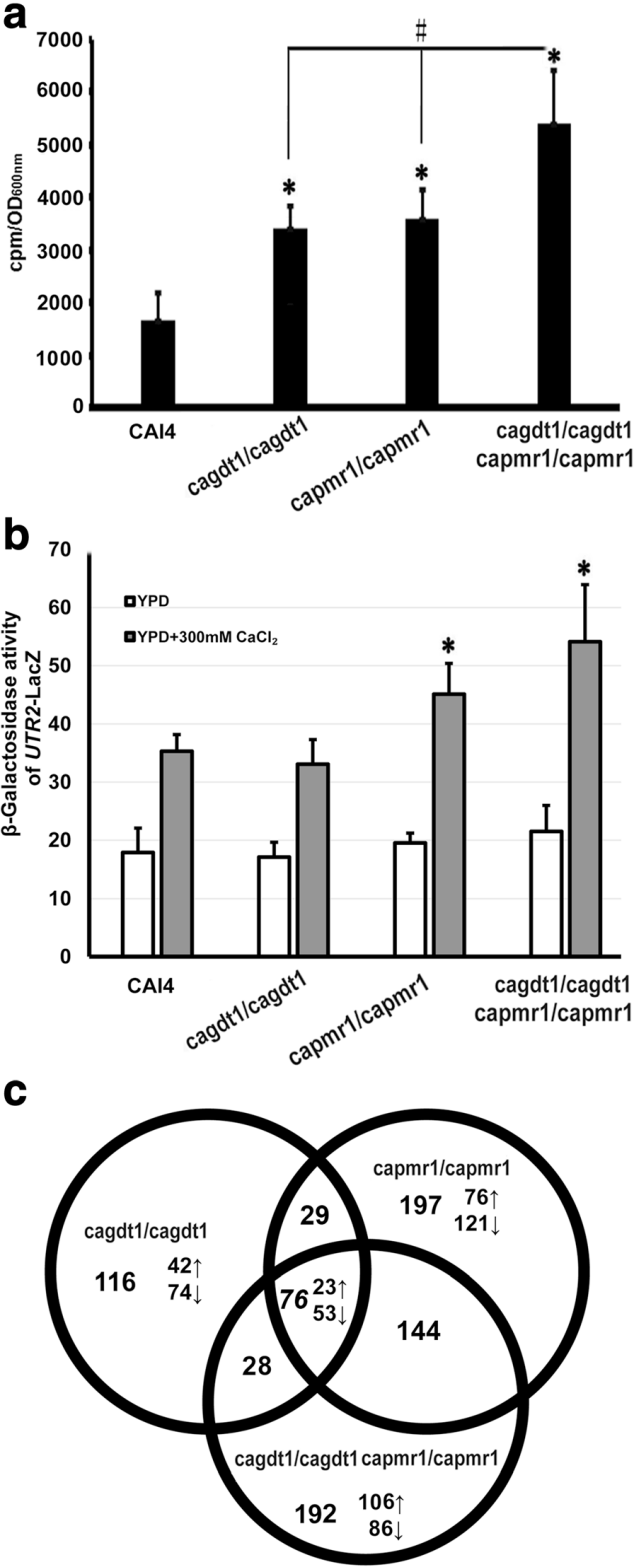
Function of *CaGDT1* and *CaPMR1* in calcium uptake, calcineurin signaling and gene expression. **a** Ca^2+^ accumulation in *C. albicans* cells lacking *CaGDT1* and/or *CaPMR1*. Cells of the wild type (CAI4), the *gdt1/gdt1* mutant, the *pmr1/pmr1* and the *gdt1/gdt1 pmr1/pmr1* mutant were grown to log-phase in YPD medium in the presence of ^45^Ca^2+^, and OD_600nm_ values and intracellular ^45^Ca^2+^ levels of these strains were measured. **b** β-galactosidase activities of *UTR2*-*lac*Z reporter in the wild type and the three mutant cells in the absence or presence of 300 mM CaCl_2_. *indicates the statistically significant differences between each mutant and the wild-type (*p <* 0.05). ^#^indicates the significant difference between the *gdt1/gdt1 pmr1/pmr1* mutant and each of its two single-gene deletion mutants (*<* 0.05). **c** Shared differentially expressed genes (DEGs) between cells lacking the *gdt1/gdt1*, the *pmr1/pmr1* or the *gdt1/gdt1 pmr1/pmr1* mutants. DEGs for each mutant are obtained from its comparison to the wild type, which include 249 (=116 + 29 + 76 + 28) genes for the *gdt1/gdt1*, 446 (=197 + 29 + 76 + 144) genes for the *pmr1/pmr1* mutant, and 440 (=192 + 28 + 76 + 144) genes for the *gdt1/gdt1 pmr1/pmr1* mutant, respectively. Total numbers of shared DEGs between two, or among three, mutants are indicated

Although deletion of *CaGDT1* did not alter the sensitivity of *C. albicans* cells to calcium stress, we showed that *CaGDT1* complemented the function of *ScGDT1* in calcium stress ([30]; Additional file 1: Figure S1A). Both ScGdt1 and its human ortholog TMEM165 are involved in the regulation of cytosolic Ca^2+^ and pH homeostasis [7]. We next measured the uptake of ^45^Ca^2+^ in mutants for *CaGDT1* and/or *CaPMR1*. As compared to the wild type, both the *gdt1/gdt1* and the *pmr1/pmr1* mutants exhibited similar high levels of ^45^Ca^2+^ uptake in YPD medium, while their double-gene mutant showed an even higher level of ^45^Ca^2^ uptake (Fig. 3a). Increased calcium uptake was also observed for cell lacking *ScPMR1* [41]. These results suggest that CaGdt1 and CaPmr1 have additive functions in the regulation of calcium homeostasis. This is consistent with observations for ScGdt1 and ScPmr1 in *S. cerevisiae* [7, 42].

We next examined activation levels of the calcium/calcineurin signaling in these mutants with the *lac*Z reporter of *CaUTR2*, a downstream target of CaCrz1 [43]. There was no significant difference in the *lac*Z activity between the wild type and each of three mutants in YPD medium (Fig. 3b). In the presence of 0.2 M CaCl_2_, both the *pmr1/pmr1* and the double-gene mutants showed higher *lac*Z activities than, but the *gdt1/gdt1* mutant showed a similar *lac*Z activity to, the wild type (Fig. 3b). These *CaUTR2*p::*lac*Z activity results agree with our transcriptomic profiling data on the transcript levels of *CaUTR2* (CAWG_02505) in these mutants and the wild type (SuppInfo 1 and 2 [GEO Accession number: GSE100737]). Taken together, these data indicate that CaPmr1 is the major controller of calcium homeostasis in *C. albicans*, which in consistent with observations on ScPmr1 in *S. cerevisiae* [7, 42].

### CaGdt1 plays a role in the CaCek1-mediated, but not CaMkc1-mediated, cell wall integrity signaling

**Fig. 4:**
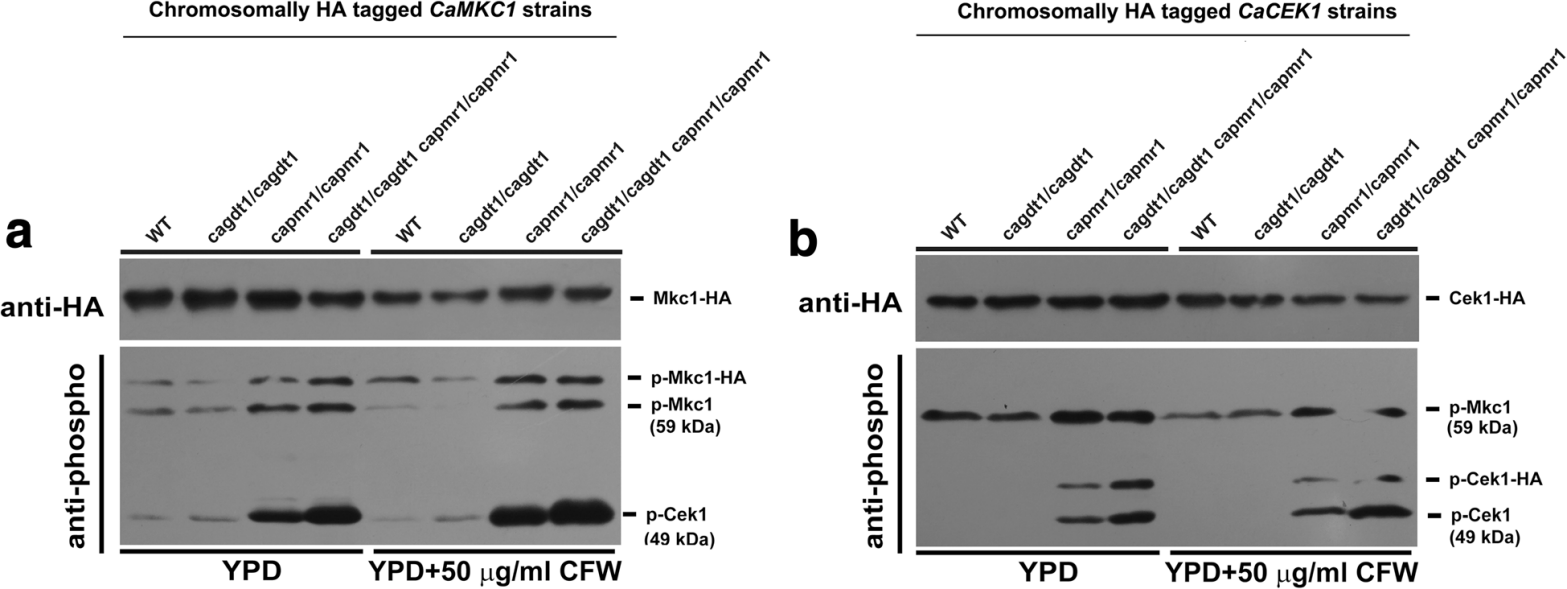
Activation of the CaMkc1- and CaCek1-mediated MAP kinase signaling in cells lacking *CaGDT1* and/or *CaPMR1*. Phosphorylation levels of CaMkc1, CaCek1 and their C-terminally HA-tagged versions were determined simultaneously by Western blotting using the Phospho-p44/42 MAPK Rabbit mAb in protein extracts prepared from log-phase growing cells of the wild-type (WT) CAI4, the WJCAG22 (*gdt1/gdt1*), the NGY98 (*pmr1/pmr1*) and the WJCAG25 (*pmr1/pmr1 gdt1/gdt1*) strains, in which either the *CaMKC1* allele (Panel **a**) or the *CaCEK1* allele (Panel **b**) was chromosomally HA-tagged, in the presence or absence of 50 μg ml^− 1^ Calcofluor white (CFW). The anti-HA antibody (Abmart, Shanghai, China) was used to reveal the total amount of CaMkc1-HA and CaCek1-HA proteins loaded. Each lane was loaded with 50 μg of total proteins

Deletion of *CaPMR1* activates the Mkc1-mediated CWI signaling [10]. Since deletion of *CaGDT1* increased the sensitivity of cells lacking *CaPMR1* to cell wall stresses (Fig. 1a), we examined the effect of *CaGDT1* deletion on CWI signaling. We chromosomally tagged the HA epitope to the C-terminus of the *CaPKC1* allele and the *CaMKC1* allele, respectively, in the wild type CAI4, the WJCAG22 (*gdt1/gdt1*), the NGY98 (*pmr1/pmr1*) and the WJCAG25 (*gdt1/gdt1 pmr1/pmr1*) strains (Additional file 1: Figure S2A, 2C and 2D). In YPD medium, phosphorylation levels of the wild type CaMkc1 (or the HA-tagged CaMkc1-HA fusion) were similar between the wild type and the *gdt1/gdt1* strains in the absence or presence of 50 μg ml^− 1^ CFW (Fig. 4a). As reported previously [10], phosphorylation levels of CaMkc1 (or CaMkc1-HA) increased with similar degrees in both the *pmr1/pmr1* and the *gdt1/gdt1 pmr1/pmr1* mutants as compared to the wild type (Fig. 4a). Similar patterns for CaMkc1 phosphorylation levels were also observed in C-terminally HA tagged *CaCEK1* strains (Fig. 4b). These results indicate that CaPmr1 does, but CaGdt1 does not, play a role in the CaMkc1-mediated CWI signaling -.

The CaCek1-mediated CWI signaling is also involved in cell wall construction [18]. Phosphorylated CaCek1 and CaCek1-HA) were not detected in both the wild type and the *gdt1/gdt1* strains, while the *gdt1/gdt1 pmr1/pmr1* mutant showed a higher level of phosphorylated CaCek1 and CaCek1-HA than the *pmr1/pmr1* mutant in the absence or presence of CFW (Fig. 4b). Similarly, in C-terminally HA tagged *CaMKC1* strains, the *gdt1/gdt1 pmr1/pmr1* mutant showed a higher level of phosphorylated CaCek1 than the *pmr1/pmr1* mutant in the absence or presence of CFW, while phosphorylated CaCek1 was detected at very low levels in both the wild type and the *gdt1/gdt1* strains, with a slightly higher level in the latter (Fig. 4a). Taken together, these data suggest that CaGdt1 and CaPmr1 are both involved in the activation of CaCek1-mediated CWI signaling independent of cell wall stress.

### CaGdt1 is localized in the Golgi apparatus but at distinct sites from CaPmr1

**Fig. 5:**
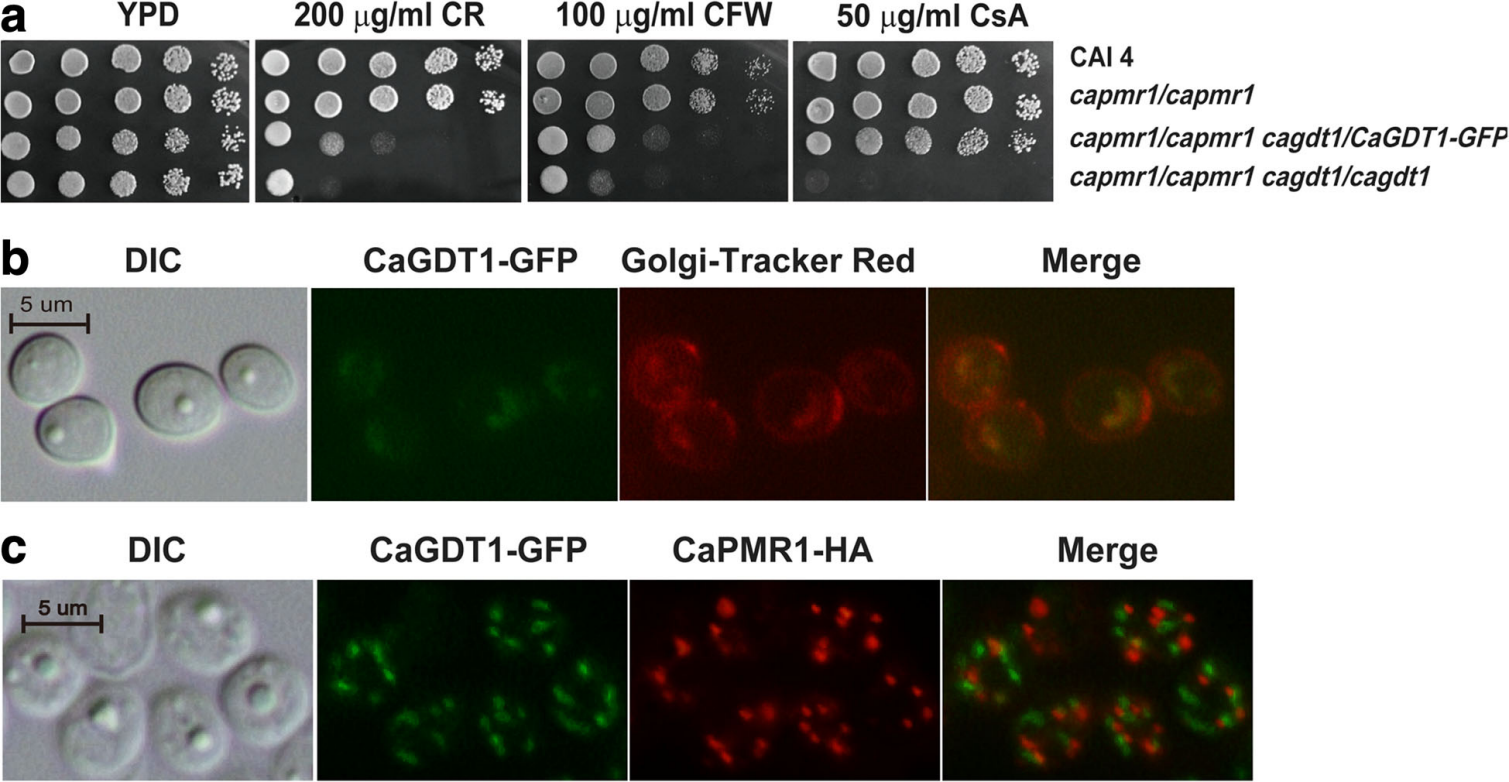
Subcellular localization of CaGdt1 and its function in virulence. **a** Functional test of the *GDT1-GFP* allele in the sensitivity of *C. albicans* cells to Congo red (CR), Calcofluor white (CFW) and cyclosporine A (CsA) in WJCA102 cells (*pmr1/pmr1 gdt1/GDT1-GFP*). **b** co-localization of CaGDT1-GFP and Golgi-Tracker Red marker in the WJCA102 cells. **c** Co-localization of CaGDT1-GFP and CaPMR1-HA in WJCA111 cells through indirect immunofluorescent approach. Images of differential interference contrast (DIC), GFP, red florescence derived from Golgi-Track Red dye (**b**) or from goat anti-mouse IgG conjugated to Alexa Fluor 555 (for CaPMR1-HA protein) (**c**) and their merged images are presented. Scale bar, 5 μm

To determine the subcellular localization of CaGdt1, we chromosomally tagged the green florescent protein (GFP) to the C-terminus of CaGdt1 in the heterozygous mutant for *CaGDT1* generating WJCA102 (CAI4 *pmr1pmr1 GDT1-GFP/gdt1*) (Additional file 1: Figure S2). Like the *pmr1/pmr1* mutant, the WJCA102 mutant was not sensitive to CsA, while the *pmr1/pmr1 gdt1/gdt1* mutant was (Fig. 5a). This indicates that the *CaGDT1-GFP* allele is functional. In log-phase growing cells of WJCA102, CaGDT1-GFP was colocalized with the Golgi Tracker Red dye (Fig. 5b).

Similarly, we constructed the WJCA111 (RM1000 *PMR1/PMR1::HA-HIS1 GDT1/GDT1::GFP-URA3*) strain to examine the colocalization between CaGdt1 and CaPmr1 (Data not shown). Through indirect immunofluorescent approach, we found that the CaGDT1-GFP protein was not colocalized with the CaPMR1-HA in log-phase growing WJCA111 cells (Fig. 5c). It should be noted that there was a discrepancy in CaGDT1-GFP localization between Fig. 5b and c, which could be due to the difference in sample processing for florescent microscopy as described in the Methods section. In response to depletion of extracellular calcium (1 mM EGTA treatment) or high levels of extracellular calcium, the CaGDT1-GFP protein was also not colocalized with the CaPMR1-HA in WJCA111 cells (Additional file 1: Figure S3). Taken together, these results indicate that CaGdt1 is present in the Golgi apparatus but at distinct sites from CaPmr1 in *C. albicans*.

### Transcriptomic profiling of cells lacking *CaGDT1* and/or *CaPMR1*

To help us further understand functions of CaGdt1 and CaPmr1, we carried out transcript profiling for the wild type CAI4, the *gdt1/gdt1*, the *pmr1/pmr1*, and the *gdt1/gdt1 pmr1/pmr1* mutants, growing in log phase in YPD medium at 30 °C (GEO accession number: GSE100737). Transcripts of 5901 genes at various expression levels were detected in each of these four strains (SuppInfo 1 [GEO Accession number: GSE100737]). As compared to the wild type, there are 249, 446 and 440 differentially expressed genes (DEGs) in the *gdt1/gdt1*, the *pmr1/pmr1* and the *gdt1/gdt1 pmr1/pmr1* mutants, respectively (Fig. 3c; SuppInfo 2). Deletion of *CaGDT1* does not affect the transcript level of *CaPMR1*, and vice versa (SuppInfo 2).

There are 105 shared DEGs between the *gdt1/gdt1* and the *pmr1/pmr1* mutants, which accounts for 42% (105/249) of total DEGs due to deletion of *CaGDT1* or 24% (105/446) of total DEGs due to deletion of *CaPMR1* (Fig. 3c). This indicates that CaGdt1 and CaPmr1 have shared and distinct functions. Furthermore, there are 76 shared DEGs among the *gdt1/gdt1*, the *pmr1/pmr1* and the *gdt1/gdt1 pmr1/pmr1* mutants, with 23 of them being up-regulated and 53 of them being down-regulated (Fig. 3c; Table 3). Using a semi-quantitative RT-PCR approach, we confirmed the transcript levels of selected 15 out of these 76 genes in the wild type, the *gdt1/gdt1*, the *pmr1/pmr1* and the *gdt1/gdt1 pmr1/pmr1* mutants (Additional file 1: Figure S4). The 23 up-regulated DEGs are involved in the cellular transport, including the transporters of long-chain fatty acids (*PXA1* and *PXA2*), the calcium channel (*CCH1*) and the potassium transporter (*TRK1*) (Table 3). Similarly, a big portion of the 53 down-regulated DEGs are also involved in the cellular transport, with different substrates including amino acids (*MUP1*, *QDR1*, *ALP1*, *GNP1*, *TPO3* and *TPO4*) [44], lipids (*PDR16*), nucleosides (*CNT*) [45], calcium ions (*RCH1*) [13] and sodium ions (*ENA2*). In addition, 10 of these down-regulated DEGs are involved in the protein synthesis (Table 3). These data suggest that core functions shared by CaGdt1 and CaPmr1 are involved in the regulation of cellular transport of metal ions and amino acids.

CaGdt1 homologs in humans and *S. cerevisiae* have been shown to regulate glycosylation pathway [25, 27]. In *S. cerevisiae*, there are two main types of glycosylation, the *N*-linked and the O-linked glycosylation (http://www.kegg.jp/kegg/pathway.html). The *N*-linked glycosylation is the attachment of the oligosaccharide known as glycan to the amide nitrogen of an asparagine residue of a protein, There are 30 genes involved in the *N*-linked glycosylation in *S. cerevisiae*, with 20 of them having homologs in *C. albicans* (Additional file 1: Table S2). Transcript levels of CAWG_02847 (*DIE2*) and CAWG_03939 (*STT3*) are up-regulated only in the *gdt1/gdt1 pmr1/pmr1* mutant, while that of CAWG_04868 (*OST3*) is up-regulated in both the *pmr1/pmr1* mutant and the *gdt1/gdt1 pmr1/pmr1* mutant, but not in the *gdt1/gdt1* mutant (Additional file 1: Table S2). The *O*-linked glycosylation is the attachment of a sugar molecule (such as N-acetyl-galactosamine, N-acetylglucosamine and mannose) to an oxygen atom in serine or threonine residue of a protein. There are 13 genes involved in the *O*-linked glycosylation n *S. cerevisiae*, with 7 of them showing homologs in *C. albicans* (Additional file 1: Table S3). Expression of CAWG_05623 (*PMT1*) and CAWG_04353 (*PMT4*) is up-regulated only in the *gdt1/gdt1 pmr1/pmr1* mutant (Additional file 1: Table S3). Furthermore, from the *C. albicans* genome database we identified 20 genes involved in the CWI pathway based on their homologs in *S. cerevisiae* (Additional file 1: Table S4) [46]. Only one gene CAWG_01201 (*SWI4*) was transcriptionally upregulated in each of three mutants, while expression of the gene CAWG_05506 (*BEM2*) was down regulated in the *pmr1/pmr1* mutant, but not affected in both the *gdt1/gdt1* mutant and the *gdt1/gdt1 pmr1/pmr1* mutant (Additional file 1: Table S4).

## Discussion

Congenital disorders of glycosylation (CDG) are rare inherited diseases causing severe growth and psychomotor retardations in patients, with most of their genetic defects affecting the glycosylation process. The human ortholog for yeast ScGdt1, TMEM165, is one of these CDG-associated Golgi proteins and might function as a Ca2^+^/H^+^ antiporter and/or a Mn^2+^ transporter in the Golgi [26, 27, 46–48]. In line with these findings, we have provided evidence that the *C. albicans* functional ortholog of ScGdt1, CaGdt1, plays a compensatory role for CaPmr1, the high affinity Ca^2+^/Mn^2+^ P-type ATPase that is required for Ca^2+^ and Mn^2+^ transport into the Golgi and involved in Ca^2+^ dependent protein sorting processing, in the regulation of calcium homeostasis and cell wall stress. In addition to the role of CaPmr1 in activation of Mkc1-mediated CWI signaling [10], we show that deletion of *CaPMR1* activates the Cek1-mediated CWI signaling (Fig. 4). This agrees with the function of Cek1 in cell wall construction [18]. In contrast, deletion of *CaGDT1* alone does not affect the Mkc1-mediated CWI signaling, but activates the Cek1-mediated signaling. Although *C. albicans* cells lacking *CaPMR1* are not sensitive to SDS [10], which targets both the cell wall and the plasma membrane, deletion of *CaGDT1* renders *C. albicans* cells lacking *CaPMR1* sensitive to SDS and more sensitive to hygromycin B (Additional file 1: Figure S1D). We also demonstrate that CaGdt1 and CaPmr1 are present at distinct sites in the Golgi apparatus, which is not affected by the calcium depletion or high levels of calcium in the extracellular environment. CaGdt1 is a functional homolog of ScGdt1 in the response of *S. cerevisiae* cells to calcium and cell wall stresses (Additional file 1: Figure S1; 30). Therefore, our data supports the hypothesis that CaGdt1 together with human TMEM165 and ScGdt1 might form a new group of Golgi-localized Ca^2+^/H^+^ exchangers [6].

As the central player in the calcium signaling, calcineurin has been shown to play a role in early synaptic dysfunction and neuronal death in mammalian cells [49]. In addition, ER stress can induce nonapoptotic cell death in yeasts, which can be blocked by the action of calcineurin [50]. Consistently, we show here that calcineurin function is required for the growth as well as the cell wall and vacuolar integrity of cells lacking both *CaGDT1* and *CaPMR1*. Therefore, the calcium/calcineurin signaling, CaGdt1 and CaPmr1 work together to regulate the integrity of both the external cell wall and the intracellular vacuole in *C. albicans*.

Deletion of *ScGDT1* increases the calcium sensitivity of *S. cerevisiae* cells lacking *ScPMR1*, while deletion of *CaGDT1* does not affect the calcium sensitivity of *C. albicans* cells lacking *CaPMR1* (Fig. 1a; Additional file 1: Figure S1A). It is interesting to note that although deletion of *CaGDT1* increases the intracellular calcium concentration in *C. albicans* cells lacking *CaPMR1* and elevates their activation levels of the calcium/calcineurin signaling, it does not affect their calcium sensitivity (Figs. 1 and 3a and b). Nevertheless, deletion of *CaGDT1* increases the sensitivity of *C. albicans* cells lacking *CaPMR1* to cell wall and ER stresses (Fig. 1). Therefore, the calcium homeostasis in the ER/Golgi apparatus is mainly involved in the regulation of the cell wall integrity. This is further supported by our recent observation on the deletion effect of *CaRCH1*, encoding a negative regulator of calcium uptake on the plasma membrane, on cells lacking *CaPMR1* [51]. In addition, normal growth of *C. albicans* cells lacking both *CaGDT1* and *CaPMR1* is, but that of *S. cerevisiae* cells lacking both *ScGDT1* and *ScPMR1* is not, sensitive to the inhibition of calcineurin function (Fig. 1b; Additional file 1: Figure S1C). Furthermore, CaGdt1 and CaPmr1 localize at different sites of the Golgi compartment in *C. albicans*, irrespective of alterations in extracellular calcium levels (Fig. 5; Additional file 1: Figure S3). In contrast, ScGdt1 and ScPmr1 are co-localized in the Golgi compartment of *S. cerevisiae* cells [6]. This difference in the relative localization between Gdt1 and Pmr1 might partially contribute to the differential response of cells lacking both *GDT1* and *PMR1* to the inhibition of calcineurin in these two microorganisms.

To further investigate the mechanism by which both CaGdt1 and CaPmr1 regulate the survival of *C. albicans* cells, we have examined the transcriptomic profiles of *C. albicans* cells lacking *CaGDT1* and/or *CaPMR1*. Cells lacking *CaGDT1* or *CaPMR1* share a significant part of DEGs (Fig. 3c), suggesting that CaGdt1 and CaPmr1 have both shared and distinct functions in *C. albicans* cells. This is consistent with their distinct localizations in the Golgi compartments of *C. albicans* cells. We have shown that 192 DEGs are exclusively present in the *gdt1/gdt1 pmr1/pmr1* mutant (Fig. 3c). Functions of these genes should be responsible for the unique phenotypes of *C. albicans* cells lacking both *CaGDT1* and *CaPMR1*. Some of these genes might be regulated by the calcium signaling, so inhibition of their transcription by CsA might be responsible for the failure of *C. albicans* cells lacking both *CaGDT1* and *CaPMR1* to survive in the presence of CsA. As compared to the wild type cells, cells lacking *CaGDT1*, *CaPMR1* or both share 76 DEGs, and most of them involved in the protein synthesis and the transport of amino acids, lipids and nucleosides are down-regulated (Table 3) This might explain why *C. albicans* cells lacking *CaGDT1* and/or *CaPMR1* grow slower than the wild type (Additional file 1: Figure S5).

**Table 3:** List of shared 76 differentially expressed genes (DEGs) due to deletion of *CaGDT1*, *CaPMR1* or both of them

Systematic name	Standard name	Systematic name	Standard name	Systematic name	Standard name	Systematic name	Standard name
Up-regulated (23)							
Metabolism							
*CAWG_02178*	*CHS2*	*CAWG_05663*	*LIP8*	*CAWG_05893*	*BLM3*	*CAWG_06046*	*IPK2*
Transcription							
*CAWG_01201*	*SWI4*	*CAWG_03966*	*SRD1*				
Cellular transport, transport facilities and transport routes							
*CAWG_00232*	*PXA2*	*CAWG_01394*	*PXA1*	*CAWG_01264*	*CCH1*	*CAWG_02805*	*HGT4*
*CAWG_00848*	*SSH1*	*CAWG_02090*	*TRK1*	*CAWG_01302*	*TUB2*		
Signal transduction mechanism							
*CAWG_04580*	*RIM21*	*CAWG_00431*	*SWE1*				
Unclassified proteins							
*CAWG_00151*	*orf19.4922*	*CAWG_04123*	*orf19.862*	*CAWG_03695*	*orf19.4706*	*CAWG_04133*	*orf19.850*
*CAWG_01315*	*orf19.6048*	*CAWG_05664*	*orf19.1344*	*CAWG_04023*	*orf19.1580*	*CAWG_04799*	*orf19.3910*
Down-regulated (53)							
Metabolism							
*CAWG_02002*	*URA2*	*CAWG_03174*	*MNN4*	*CAWG_06095*	*GPD1*		
Transcription							
*CAWG_03683*	*MDN1*	*CAWG_04089*	*STP4*				
Protein synthesis							
*CAWG_00892*	*REI1*	*CAWG_05130*	*NIP7*	*CAWG_05006*	*NOG2*	*CAWG_05890*	*NSA1*
*CAWG_03515*	*HCA4*	*CAWG_05685*	*ENP2*	*CAWG_05072*	*MRT4*	*CAWG_06009*	*PES1*
*CAWG_04963*	*SPB1*	*CAWG_05748*	*UTP18*	*CAWG_02769*	*MAK21*		
Cellular transport, transport facilities and transport routes							
*CAWG_00254*	*MUP1*	*CAWG_01758*	*QDR1*	*CAWG_01009*	*PDR16*	*CAWG_05897*	*SIT1*
*CAWG_03747*	*RCH1*	*CAWG_01334*	*ENA2*	*CAWG_05173*	*YHM1*	*CAWG_00353*	*ALP1*
*CAWG_04346*	*CNT*	*CAWG_01729*	*TPO4*	*CAWG_05325*	*GNP1*	*CAWG_00547*	*TPO3*
Signal transduction mechanism							
*CAWG_01171*	*SHA3*	*CAWG_02761*	*RAS2*				
Unclassified proteins							
*CAWG_00174*	*orf19.6355*	*CAWG_03036*	*orf19.6770*	*CAWG_05031*	*orf19.5628*	*CAWG_05230*	*orf19.3449*
*CAWG_00337*	*orf19.2319*	*CAWG_03114*	*orf19.3089*	*CAWG_05139*	*orf19.3469*	*CAWG_05320*	*orf19.1200*
*CAWG_00416*	*orf19.4886*	*CAWG_03529*	*orf19.2730*	*CAWG_05144*	*orf19.3463*	*CAWG_05329*	*orf19.1189*
*CAWG_00694*	*orf19.4445*	*CAWG_04078*	*orf19.5785*	*CAWG_05179*	*orf19.3393*	*CAWG_05461*	*orf19.7011*
*CAWG_00989*	*orf19.4479*	*CAWG_04724*	*orf19.6660*	*CAWG_05189*	*orf19.3406*	*CAWG_06060*	*orf19.1363*
*CAWG_01012*	*orf19.1030*	*CAWG_05000*	*orf19.5747*	*CAWG_05229*	*orf19.3448*		

During N-linked glycosylation, an oligosaccharide chain is assembled on the carrier molecule dolichyl pyrophosphate in the following order: 2 molecules of N-acetylglucosamine (GlcNAc), 9 molecules of mannose, and 3 molecules of glucose. *DIE2* encodes an alpha-1,2 glucosyltransferase that catalyzes the addition of the third glucose moiety [52]. This 14-residue oligosaccharide core is then transferred by the oligosaccharyl transferase complex (OST complex), consisting of nine ER integral membrane protein subunits Ost1–6, Stt3, Swp1 and Wbp1, to an asparagine residue on a nascent prolylpeptide in the ER. As this protein progresses through the Golgi apparatus, its oligosaccharide core is modified by trimming or extension. As a result, a diverse array of N-glycosylated proteins is generated [52, 53]. *STT3* is an essential gene, and its conditional mutants are defective in cell wall biosynthesis [54]. On the other hand, protein O-mannosylation (POM) is an evolutionarily conserved, essential posttranslational modification that impacts a variety of cellular processes in both fungi and mammals, which is initiated at the ER by a family of dolichyl phosphate mannose-dependent protein O-mannosyltransferases (PMTs). PMTs transfer mannose residues from dolichyl phosphate-D-mannose to protein seryl/threonyl residues. The POM is essential for yeast cell wall integrity [55, 56]. In *S. cerevisiae*, the PMT family is highly redundant (Pmt1–Pmt6) and is classified into three subfamilies, PMT1, PMT2, and PMT4, which mannosylate distinct target proteins [56]. In the present study, we have shown that lack of *CaGDT1* and *CaPMR1* upregulates the expression of three N-linked glycosylation genes *CaDIE2*, *CaOST3* and *CaSTT3* as well as two major O-linked glycosylation genes *CaPMT1* and *CaPMT4* (Additional file 1: Tables S2 and S4). Since the activation level of the calcium/calcineurin signaling is significantly elevated due to deletion of *CaGDT1* and *CaPMR1*, our current results indicate that expression of these six glycosylation genes might be under control of the transcription factor CaCrz1. In this way, the defect in cell wall integrity due to deletion of *CaGDT1* and *CaPMR1* could be compensated by enhanced glycosylation process, specifically the increased biosynthesis of the cell wall beta1,6-glucan [54].

Chitin is an essential component of the fungal cell wall, and *C. albicans* has four chitin synthases, two class I enzymes encoded by *CaCHS2* and *CaCHS8*, one class II enzyme encoded by *CaCHS1* and one Class IV enzyme encoded by *CaCHS3*. All four genes are stimulated by CFW treatment. In addition, only expression of *CaCHS2* and *CaCHS8* is mediated by CaMkc1-mediated CWI signaling [57]. Our transcript profiling data indicates that expression of both *CHS1* (CAWG_05611) and *CHS8* (CAWG_02407) was not affected due to deletion of *CaGDT1* and/or *CaPMR1*. *CHS2* (CAWG_02178) was up regulated in all three mutants, while *CHS3* (CAWG_00138) was only up regulated in the *pmr1/pmr1* and the *gdt1/gdt1 pmr1/pmr1* mutants (SuppInfo 2 [GEO Accession number: GSE100737]). In addition, we have observed that expression of *CaSWI4* is upregulated due to deletion of *CaGDT1* and/or *CaPMR1*, while expression of *BEM2* is down regulated due to the deletion of *CaPMR1* (Additional file 1: Table S4). *CaSWI4* encodes one of terminal transcription factors of the CWI pathway and its mutants are sensitive to the cell wall-interfering agent caffeine [58]. Bem2 is a negative regulator of the central CWI regulator Rho1 in *S. cerevisiae*, albeit not characterized in *C. albicans* [46]. These results agree with our observations on the activation of Mkc1- and Cek1-mediated CWI signaling in these mutants (Fig. 4) as well as the previous study [57]. Since Ca^2+^-induced upregulation of *CHS* promoters is independent of the CWI signaling [57], inhibition of expression of both *CaCHS2* and *CaCHS3* by CsA might contribute to defects in the integrity of the external cell wall and intracellular vacuoles of *C. albicans* cells lacking both *CaGDT1* and *CaPMR1*, leading to their death.

## Conclusions

In this study, we have demonstrated that CaPmr1 is the major player in the regulation of calcium homeostasis and cell wall stress, while CaGdt1 plays a compensatory role for CaPmr1 in the Golgi compartment in *C. albicans*. It is interesting to note that mammalian TMEM165/Gdt1 is regulated at posttranslational level and directed from the Golgi to lysosomes for degradation in response to high Mn^2+^ concentrations [48]. In contrast, expression of *ScPMR1* is regulated at the transcriptional level, positively mediated by the transcription activator Crz1 through the calcium/calcineurin signaling pathway and negatively mediated by the transcription repressor Nrg1 through the Rim101/Nrg1 pathway [7]. In this way, Pmr1 functions as a major sensor for intracellular Ca^2+^ and Mn^2+^ concentrations to elicit a relatively slow and profound response to a change in intracellular calcium homeostasis, while Gdt1 acts as a fast modulator for the ion homeostasis in the Golgi. This combination of temporal (Pmr1) and spatial (Gdt1) regulation of key players in the Golgi might be an efficient way to control the complicated glycosylation process in eukaryotes.

## Additional file

Additional file 1: **Figure S1.** Functions of *ScGDT1* and *CaGDT1*. **Figure S2.** Strategies for chromosomally tagging GFP to the C-terminus of CaGdt1 and tagging HA to the C-terminus of CaPmr1. **Figure S3.** Co-localization of CaGDT1-GFP and CaPMR1-HA in WJCA111 cells through indirect immunofluorescent approach in response to calcium stress. **Figure S4.** Semi-quantitation of expression levels of selected 15 genes by RT-PCR. **Figure S5** Growth assay of the wild type CAI4, the *gdt1/gdt1*, the *pmr1/pmr1* and the *gdt1/gdt1 pmr1/pmr1* mutants. **Table S1.** Primers used in this study. **Table S2.** Genes involved in the N-linked glycosylation process. **Table S3.** Genes involved in the O-linked glycosylation process. **Table S4.** List of 20 genes involved in the cell wall integrity pathway in *Candida albicans*. (PDF 2978 kb)

## Abbreviations

BSA: bovine serum albumin
CDG: congenital disorders of glycosylation
CFW: calcofluor white
CR: Congo red
CsA: cyclosporine A
CWI: cell wall integrity
ER: endoplasmic reticulum
GFP: green florescent protein
HEPES: hydroxyethyl piperazineethanesulfonic acid
MAPK: mitogen-activated protein kinase
PBS: phosphate buffer saline
PCR: polymerase chain reaction
RPKM: reads per kilobase million
RT: reverse transcription
TMEM165: transmembrane protein 165
UPF0016: uncharacterized protein family UPF0016
YPD: yeast peptone dextron
