# Supplementary material for: CaGdt1 plays a compensatory role for the calcium pump CaPmr1 in the regulation of calcium signaling and cell wall integrity signaling in Candida albicans
Source: Cell Commun Signal. 2018 Jun 28;16:33. doi: 10.1186/s12964-018-0246-x (PMC6025805; doi:10.1186/s12964-018-0246-x)
Supplement: Supplementary file 1 — Figure S1. Functions of ScGDT1 and CaGDT1. Figure S2. Strategies for chromosomally tagging GFP to the C-terminus of CaGdt1 and tagging HA to the C-terminus of CaPmr1. Figure S3. Co-localization of CaGDT1-GFP and CaPMR1-HA in WJCA111 cells through indirect immunofluorescent approach in response to calcium stress. Figure S4. Semi-quantitation of expression levels of selected 15 genes by RT-PCR. Figure S5 Growth assay of the wild type CAI4, the gdt1/gdt1, the pmr1/pmr1 and the gdt1/gdt1 pmr1/pmr1 mutants. Table S1. Primers used in this study. Table S2. Genes involved in the N-linked glycosylation process. Table S3. Genes involved in the O-linked glycosylation process. Table S4. List of 20 genes involved in the cell wall integrity pathway in Candida albicans. (PDF 2978 kb) [file 12964_2018_246_MOESM1_ESM.pdf]

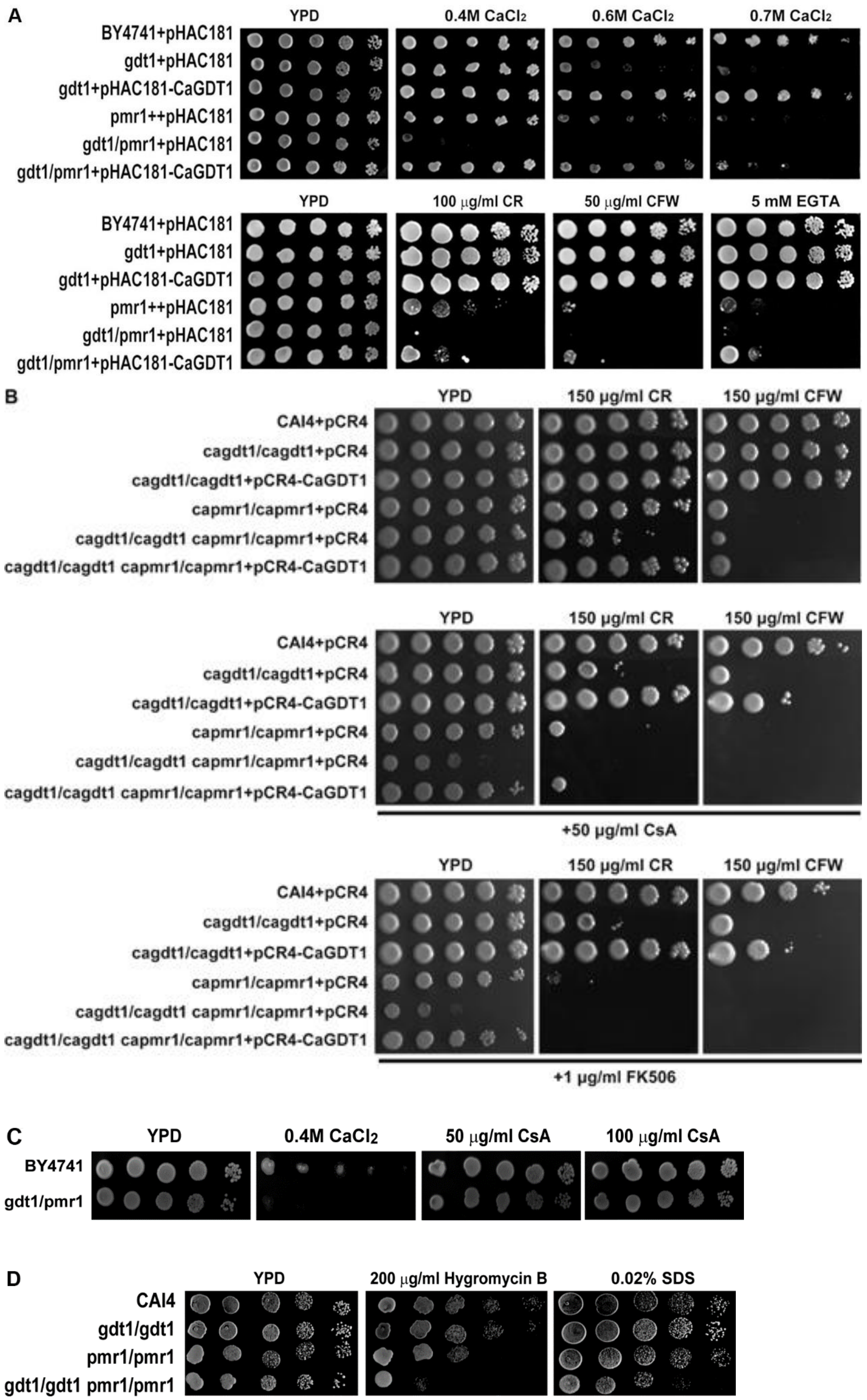

A
YPD
0.4M CaCl2
0.6M CaCl2
0.7M CaCl2
BY4741+pHAC181
gdt1+pHAC181
gdt1+pHAC181-CaGDT1
pmr1++pHAC181
gdt1/pmr1+pHAC181
gdt1/pmr1+pHAC181-CaGDT1
YPD
100 μg/ml CR
50 μg/ml CFW
5 mM EGTA
B
YPD
150 μg/ml CR
150 μg/ml CFW
CAI4+pCR4
cagdt1/cagdt1+pCR4
cagdt1/cagdt1+pCR4-CaGDT1
capmr1/capmr1+pCR4
cagdt1/cagdt1 capmr1/capmr1+pCR4
cagdt1/cagdt1 capmr1/capmr1+pCR4-CaGDT1
+50 μg/ml CsA
+1 μg/ml FK506
C
YPD
0.4M CaCl2
50 μg/ml CsA
100 μg/ml CsA
BY4741
gdt1/pmr1
D
YPD
200 μg/ml Hygromycin B
0.02% SDS
CAI4
gdt1/gdt1
pmr1/pmr1
gdt1/gdt1 pmr1/pmr1

Figure S1. Functions of *ScGDT1* and *CaGDT1*. (A), functional complementation of *ScGDT1* by *CaGDT1* in the sensitivity of budding yeast cells to cell-wall and calcium stresses. BY4741, the haploid wild-type budding yeast strain. Six indicated strains containing the pHAC181 vector or the pHAC181-CaGDT1 recombinant plasmid were grown overnight in SD-LEU medium, serially diluted by 10 times and spotted onto YPD plates in the absence or presence of 100 μg/ml Congo red (CR), 50 μg/ml Calcoflor white (CFW) red, 5 mM EGTA, 0.4M $CaCl_2$, 0.6M $CaCl_2$ or 0.7M $CaCl_2$. (**B**), phenotypes of the *Candida albicans* single-gene *cagdt1/cagdt1* and *capmr1/capmr1* mutants as well as the double-gene *cagdt1/cagdt1 capmr1/capmr1* mutant in the sensitivity to cell-wall perturbing agents in the absence or presence of cyclosporine A (CsA) or FK506. (**C**), growth of the wild-type (BY4741) and the *gdt1/pmr1* mutant in the presence of 0.4M CaCl2 or CsA as indicated. Phenotypes of the *C. albicans* single-gene *gdt1/gdt1* and *pmr1/pmr1* mutants as well as their double-gene *gdt1/gdt1 pmr1/pmr1* mutant in the sensitivity to hygromycin B and SDS (**D**). *C. albicans* strains were grown overnight, serially diluted by 10 times and spotted onto YPD plates in the absence or presence of indicated reagents.

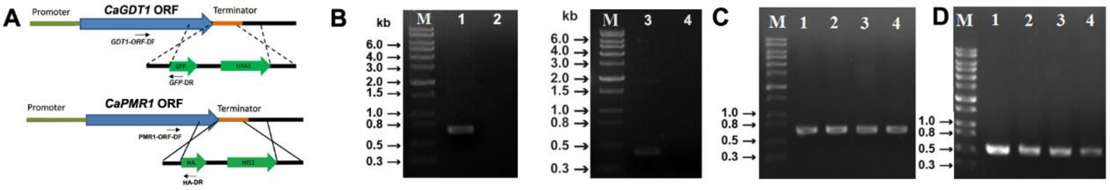

Figure S2. A, strategies for chromosomally tagging GFP to the C-terminus of CaGdt1 (upper panel) and tagging HA to the C-terminus of CaPmr1 (lower panel). Similar strategies were used for chromosomally tagging HA to the C-terminus of CaMkc1 or CaCek1 in strains described below. **B**, PCR confirmation of correct integration of the GFP-URA3 cassette in the genome of WJCA102 (CAI4 *pmr1::hisG/pmr1::hisG gdt1::FRT/GDT1::GFP-URA3*) (left panel) and the HA-HIS1 cassette in the genome of WJCA110 (RM1000 *PMR1/PMR1::HA-HIS1*) (Right panel). M, DNA size marker. A 684-bp PCR product was amplified with primers GDT1-ORF-DF and GFP-DR from the genomic DNA sample of WJCA102 (CAI4 *pmr1::hisG/pmr1::hisG gdt1::FRT /GDT1:: GFP-URA3*) (Lane 1), but not from that of the wild type CAI4 (Lane 2). A 411-bp PCR product was amplified with primers PMR1-ORF-DF and HA-DR from the genomic DNA sample of WJCA111 (RM1000 *PMR1/PMR1::HA-HIS1 GDT1/GDT1::GFP-URA3*) (Lane 3), but not from that of the wild type RM1000 (Lane 4). **C**, PCR confirmation of correct integration of the HA-URA3 cassette at the C-terminus of CaMKC1. A 735–bp fragment was amplified with primer pair MKC1-DF and HA-DR from the genomic DNA samples of the wild type WJCA201 (*MKC1/MKC1::HA-URA3*) (Lane 1), WJCA202 (CAI4 *gdt1::hisG/gdt1::FRT MKC1/MKC1::HA-URA3*) (lane 2), WJCA203 (CAI4 *pmr1::hisG/pmr1::hisG MKC1/MKC1::HA-URA3*) (Lane 3) and WJCA204 (CAI4 *pmr1::hisG/pmr1::hisG gdt1::hisG/gdt1::FRT MKC1/MKC1:: HA-URA3*) (Lane 4). **D**, PCR confirmation of correct integration of the HA-URA3 cassette at the C-terminus of CaCEK1. A 530–bp fragment was amplified with primer pair CEK1-DF and HA-DR from the genomic DNA samples of the wild type WJCA205 (Lane 1), WJCA206 (Lane 2), WJCA207 (Lane 3) and WJCA208 (Lane 4).

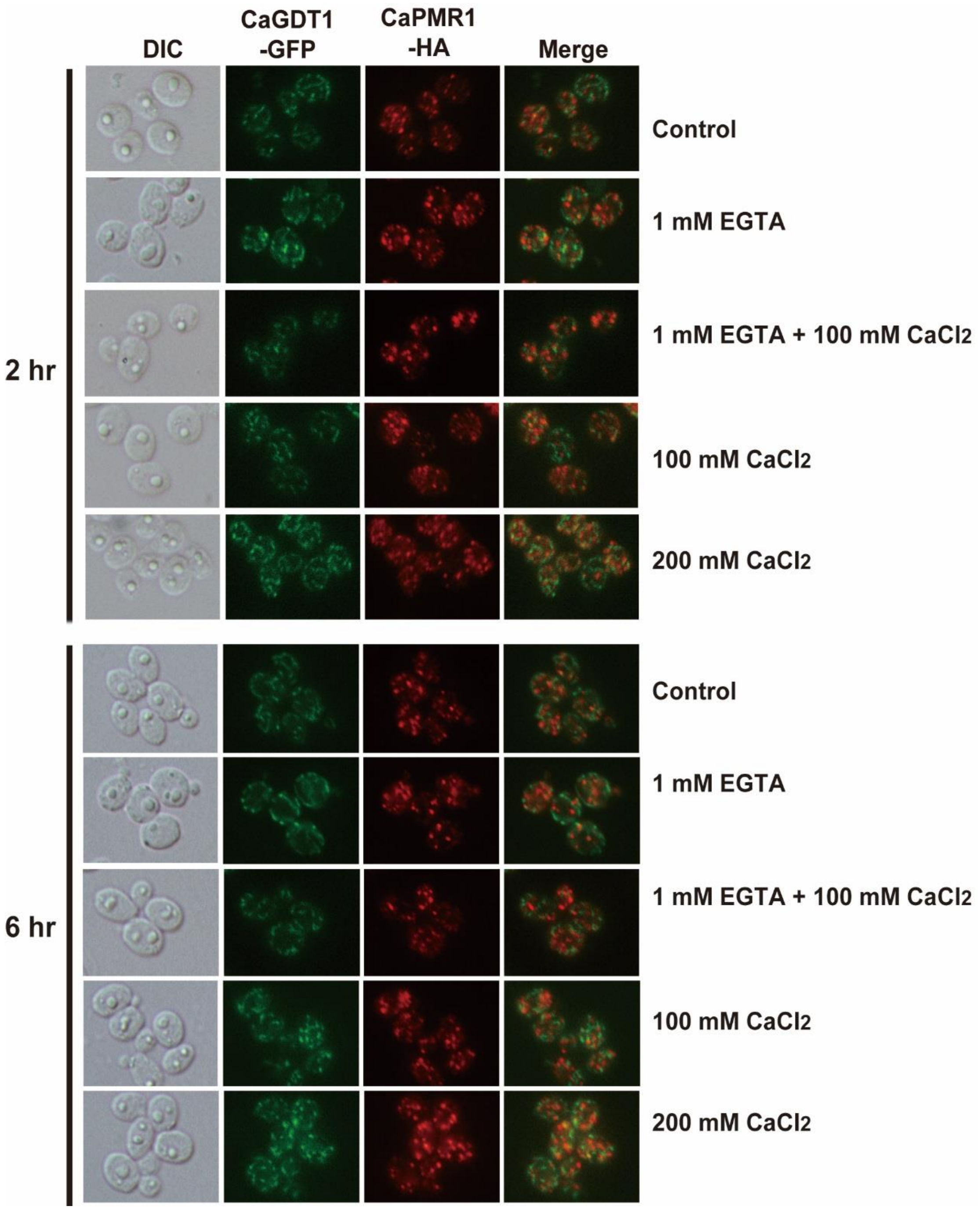

Figure S3. Co-localization of CaGDT1-GFP and CaPMR1-HA in WJCA111 cells through indirect immunofluorescent approach in response to calcium stress. Images of differential interference contrast (DIC), GFP, red florescence derived from goat anti-mouse IgG conjugated to Alexa Fluor 555 (for CaPMR1-HA protein) and their merged images are presented. Log-phase growing WJCA111 cells were incubated for 2 hr and 6 hr, respectively, at 30 ºC in the absence (control) or presence of indicated chemical agents.

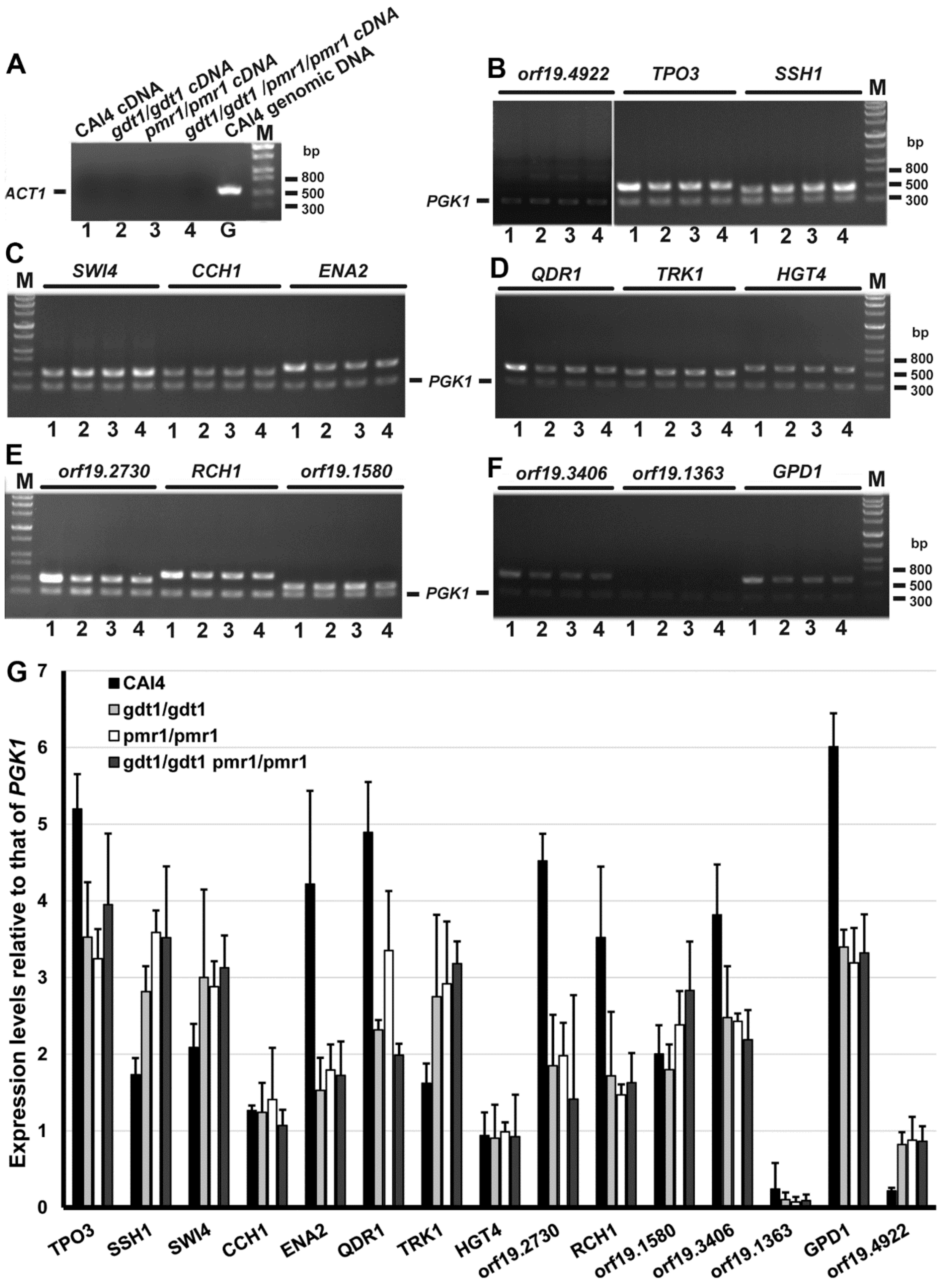

Figure S4. Semi-quantitation of expression levels of selected 15 genes in the wild type CAI4 (lane 1), the *gdt1/gdt1* (lane 2), the *pmr1/pmr1* (lane 3), the *gdt1/gdt1 pmr1/pmr1* (lane 4) mutants by RT-PCR. **A**, cDNA samples from four strains were examined for genomic DNA contamination with the primer pair CaACT1-g/m-DOWN and CaACT1-g-UP that is located within the intron of *CaACT1*. CAI4 genomic DNA was used as a positive control (lane G). **B-F**, RT-PCR products from four strains with the name of each target gene or orf indicated above the agarose gel. Primers for internal control gene

CaPGK1 were included in the PCR reaction of each target gene. The PCR product amplified from cDNAs of *CaPGK1* was indicated with a short bar, the DNA band above which was derived from cDNAs of each target gene. M, DNA size marker. **G**, DNA band intensities in agarose gels of (B-F) were quantified, and expression levels of each target gene was normalized to that of *CaPGK1*. Data were the average of three independent RT-PCR experiments.

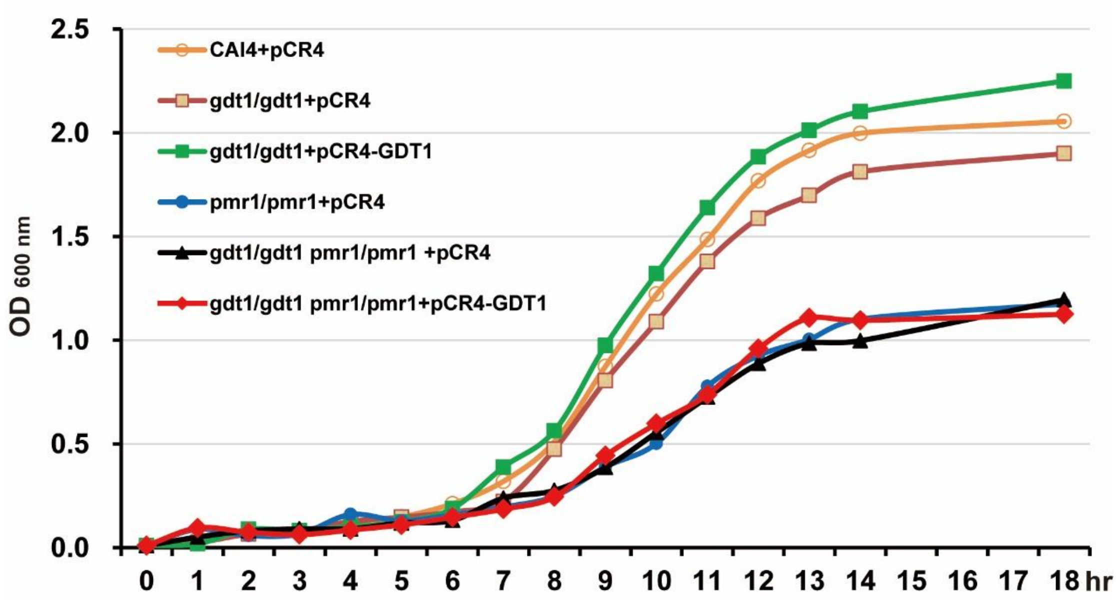

Figure S5. Growth assay of the wild type CAI4, the *gdt1/gdt1*, the *pmr1/pmr1* and the *gdt1/gdt1 pmr1/pmr1* mutants containing the pCR4 vector or the pCR4-GDT1 plasmid indicated. Cells were cultured in SD-URA medium at 30ºC for indicated hours. Data were the average of three independent experiments.

Table S1. Primers used in this study

Primer name	Sequence (5’ to 3’)	Restriction site
CaACT1-g-UP	gatttcccggctcactatg	
CaACT1-m-UP	gagatacccaattgaacacgg	
CaACT1-g/m-DOWN	ggaaacgtagaaagctggaac	
CaGDT1-GFP-DOWN	tctgatctatatagagattgtactagataaggaagaaactcaacatatgaatatgtgtgtatatgttatactt<br>gaatctaataaatatagttacaacaactctagaaggaccacctttgattg	
CaGDT1-GFP-UP	tggtggagccattgcatttttatttttcaattttgtatttttatgatgcttattatggtattgaaagtggtggtg<br>gttctaaaggtgaagaattatt	
CaGDT1-RT-DOWN	gatctatcaccccattcacc	
CaGDT1-RT-UP	ctgagggagcaactaactc	
NAT-R	gctggagcattaacattggt	
NAT-Rev	gtgccgtgcaagtttctatc	
CaPMC1-RT-DOWM	cttctggtgatgatcttgc	
CaPMC1-RT-UP	caggagtcactgttcgtatg	
CaPMR1-RT-DOWN	gtcaatacagcatggtctcc	
CaPMR1-RT-UP	caagagatgggttgagagtg	
CaVCX1-RT-DOWN	cagcacagaacgataccaag	
CaVCX1-RT-UP	gtggtgtttccgttgtcttg	
CEK1-DF	ggtagacctttattccctgg	
CEK1-HA-DOWN	cagtgttatcaaagtattaaagatattttacattaattctacttctttttcttttcaaatatcaacaaaatctattc<br>ttcacacatgtttacttaacttcctagaaggaccacctttg	
CEK1-HA-UP	taaaatcccagaagatttttcgattttgataaaatgaaagatcaattaacaattgaagatttgaaaaaatt<br>gttatatgaagagattatgaaaccattacccgggtacccatacgatgttc	
GDT 1-L-F	cgcgagctcgtaatagaggcacctgtttc	*Sac*I
GDT 1-L-R	cggggtaccgagggtcttttaggattg	*Kpn*I
GDT 1-R-F	cgcggatccctgctgcttgtattggtg	*Bam*HI
GDT 1-R-R	aactgcagggtgtcttgccaaaattc	*Pst*I
GDT1 (BamHI) –UP	cgcggatccgtaatagaggcacctgtttcg	*Bam*HI
GDT1 (BamHI)–DOWN	cgcggatccgaagaagaacaagatggtgg	*Bam*HI
GDT1 (KpnI) –UP	cggggtaccccaccttgatggtttgattg	*Kpn*I
GDT1 (SphI) –DOWN	acatgcatgcgcatgcgaagaagaacaagatggtgg	*Sph*I
GDT1-DF	gttgtcgttaaggtttgtgt	
GDT1-DR	atcaatgggtacaatggtg	
GDT1-NAT-DOWN	acatatctattgttgtttctgatctatatagagattgtactagataaggaagaaactcaacatatgaatatgt<br>gtgtatatgttatacttgaatctaataatacgactcactataggg	
GDT1-NAT-UP	catcaatcaatcaaccaatttcattttcatttcaatcctaaaagaccctctttattaataatcattacaaactc<br>ccctttttctttcttctttcttcttaaattaaccctcactaaaggg	
GDT1-ORF-DOWN	gatctatcaccccattcacc	
GDT1-ORF-UP	gctgcattaatggccatg	
GFP-DR	gccgtttcatatgatctgg	
HA-DR	ggatatcctgcatagtcc	
MKC1-DF	ctatgtcagatctttgccg	
MKC1-HA-DOWN	ggtaaaatagattcttttcatctagacgaatccacactttggtgctttgccaaaagcggtgacctatttag<br>atatagtatattaacttagtagttgattgctagaaggaccacctttg	

MKC1-HA-UP	catggctaaaccatcaggagaagagtatataaagctagaggaagagcttgggtttggattagatggtg ctatgtttaacaactactgtaacgaccaccagcccgggtacccatacgatgttc	
PMR1-DF	tcaagacgtcacggcaatg	
PMR1-DR	gtcggtgatgtctatagtg	
PMR1-HA-HIS-DOWN	gccccgcgcaattacaacataagcgtaattaaatatctcggaaaagaaaatgtttttccaatgtagattc ttgctactgattgaaaacctctggatttgaggtgtgtacatcaaggtggtag	
PMR1-HA-HIS-UP	gtttggttattttgacaagtactgtatttgctgtggatgaaattagaaagtggttacgtagaaggaaaact gtgtataccaataactatagttatggtgtacccgggtacccatacgatgttc	
PMR1-ORF-DOWN	gtcaatacagcatggtctcc	
PMR1-ORF-UP	gctcagacaaaaccggtac	
CaADH1-F	gc tctaga tgccgtaaactatctcca	
CaADH1-RHB-R	gtaacaagtatggatggagatgttgttgttgttgtatgac	
CaBRG1-RH-F	gtcatacaacaacaacaacatctccatccatacttgttac	
CaBRG1-R	aactgcagctttcggtttgatccagc	
CaADH1-RHN-R	cttaatgaaactagcagggatgttgttgttgttgtatgac	
CaNRG1-RH-F	gtcatacaacaacaacaacatccctgctagtttcattaag	
CaNRG1-R	aactgcagaatacaaaggcggcaggg	
RP10-F	ctcaaaacgtaatcgtcggaag	
**Primers for RT-PCR**		**PCR product size (bp) and gene name#**
PGK1-RT-F	cattagctccagttgctactg	315
PGK1-RT-R	gaacttcgagaccaaccatag	
CAWG_00151-F	ccaagctaaaggactctcaagg	678
CAWG_00151-R	ctgaatccagtgatagcacc	*orf19.4922*
CAWG_00547-F	ccagccagatcttgaagcac	535
CAWG_00547-R	ccaaagcaaaggaccaacac	*TPO3*
CAWG_00848-F	gtttgcctttactgtggttg	468
CAWG_00848-R	gcaacaccataacctaatcc	*SSH1*
CAWG_01201-F	gtgaccctccacgaagatac	510
CAWG_01201-R	gctgctgttgttgttgttgc	*SWI4*
CAWG_01264-F	gctatcacgtgtgcgagaac	508
CAWG_01264-R	cgatgatgcaggtccatctg	*CCH1*
CAWG_01334-F	gtgatactgtgcctgctgatc	590
CAWG_01334-R	catgacttgtgctccaactg	*ENA2*
CAWG_01758-F	gtgttgctggtgatatctgcac	527
CAWG_01758-R	gaagcaggcgataccttgag	*QDR1*
CAWG_02090-F	ctctgcgtggtgatgagatc	485
CAWG_02090-R	gtctcctttcaccttcacc	*TRK1*
CAWG_02805-F	atggctgagaggttcagtgg	579
CAWG_02805-R	ggtcgtctaccaacgacatc	*HGT4*
CAWG_03529-F	gcaccagggatataccaactg	497
CAWG_03529-R	gcgtagaactcttcgtgctg	*orf19.2730*
CAWG_03747-F	gtggattatccatgtccacc	592
CAWG_03747-R	ctgacactggtgaacgcatg	*RCH1*
CAWG_04023-F	gatgacatagaggacacgtc	428
CAWG_04023-R	gcatgccataccatgattacg	*orf19.1580*

CAWG_05189-F	gacgtgctagtcatcaactc	605
CAWG_05189-R	cctactgatattggtgcacc	*orf19.3406*
CAWG_06060-F	cgtctcatctcctagtgagac	525
CAWG_06060-R	ggtgaagatgaagaatggcc	*orf19.1363*
CAWG_06095-F	ccagatattgttactgccg	574
CAWG_06095-R	ctacaccagcagattcagc	*GPD1*

[#]Names of genes or orfs corresponding to their systemic names are presented under the sizes of PCR products.

Table S2. Genes involved in the N-linked glycosylation process

**Organisms**				**DEGs in *C. albicans* cells lacking *CaDGT1*, *CaPMR1* or *both***		
***Saccharomyces cerevisiae* (30)**		***Candida albicans* (20)**		***gdt1/gdt1***	***pmr1/pmr1***	***gdt1/gdt1 pmr1/pmr1***
Systemic name	Standard name	Systemic name	Standard name			
YMR013C	*SEC59*	CAWG_02592	*SEC59*			
YBR243C	*ALG7, TUR1*	CAWG_05888	*ALG7*			
YPL227C	*ALG5*	No				
YGL047W	*ALG13*	No				
YBR070C	*ALG14*	No				
YPR183W	*DPM1, SED3*	No				
YIL102C-A		No				
YBR110W	*ALG1*	CAWG_03225	*ALG1*			
YGL065C	*ALG2*	CAWG_04976	*ALG2*			
YNL048W	*ALG11*	CAWG_05140	*ALG11*			
YBL082C	*ALG3, RHK1*	No				
YNL219C	*ALG9*	CAWG_00691	*ALG9*			
YNR030W	*ALG12, ECM39*	CAWG_05631	*ECM39*			
YOR002W	*ALG6*	CAWG_01987	*ALG6*			
YOR067C	*ALG8, YOR29-18*	CAWG_02517	*ALG8*			
**YGR227W**	***DIE2, ALG10***	**CAWG_02847**	***DIE2***			**up**
**YGL022W**	***STT3***	**CAWG_03939**	***STT3***			**up**
YJL002C	*OST1, NLT1*	CAWG_02863	*OST1*			
YMR149W	*OST1*	CAWG_02863	*OST1*			
YOR103C	*OST2*	CAWG_06091	*OST2*			

YML019W	*OST6*	CAWG_02952	*OST6*		
**YOR085W**	***OST3***	**CAWG_04868**	***OST3***	**up**	**up**
YEL002C	*WBP1*	CAWG_00317	*WBP1*		
YDL232W	*OST4*	CAWG_06063	*OST4*		
YGL226C-A	*OST5*	No			
YGR036C	*CAX4, CWH8*	CAWG_01158	*CAX4, CWH8*		
YGL027C	*CWH41, DER7, GLS1*	No			
YBR229C	*ROT2, GLS2*				
YJR131W	*MNS1*	CAWG_01018	*MNS1*		
YLR057W	*MNL2*	No			

"No" indicates there is no homologous sequence for a *S. cerevisiae* gene in the genome of C. albicans

Table S3. Genes involved in the O-linked glycosylation process

**Organisms**				**DEGs in *C. albicans* cells lacking *CaDGT1*, *CaPMR1* or *both***		
***Saccharomyces cerevisiae* (13)**		***Candida albicans* (7)**		***gdt1/gdt1***	***pmr1/pmr1***	***gdt1/gdt1 pmr1/pmr1***
Systemic name	Standard name	Systemic name	Standard name			
YOR321W	*PMT3*	No				
**YDL095W**	***PMT1***	**CAWG_05623**	***PMT1***			**up**
YDR307W	*PMT7*	No				
YAL023C	*PMT2, FUN25*	No				
**YJR143C**	***PMT4***	**CAWG_04353**	***PMT4***			**up**
YGR199W	*PMT6*	CAWG_03338	*PMT6*			
YDL093W	*PMT5*	No				
YBR205W	*KTR3*	No				
YOR099W	*KTR1*	No				
YDR483W	*KRE2*	CAWG_02512	*MNT1*			
YER001W	*MNN1*	CAWG_04645	*MNN1*			
YGL257C	*MNT2*	CAWG_02514	*MNT2*			
YIL014W	*MNT3*	CAWG_01858	*MNT3*			

"No" indicates there is no homologous sequence for a *S. cerevisiae* gene in the genome of C. albicans

Table S4. List of 20 genes involved in the cell wall integrity pathway in *Candida albicans*

		DEGs in *C. albicans* cells lacking *CaDGT1, CaPMR1* or *both*		
Systemic name	Standard name	*gdt1/gdt1*	*pmr1/pmr1*	*gdt1/gdt1 pmr1/pmr1*
CAWG_02738	*WSC1*			
CAWG_05101	*WSC2*			
CAWG_02738	*WSC3*			
No	*ROM2*			
CAWG_00946	*TUS1*			
CAWG_02007	*STT4*			
CAWG_02450	*MSS4*			
CAWG_01622	*RHO1*			
CAWG_05506	*BEM2*		down	
CAWG_05365	*SAC7*			
CAWG_00200	*PKH1*			
CAWG_00200	*PKH2*			
CAWG_05323	*PKH3*			
CAWG_02760	*PKC1*			
CAWG_05633	*BCK1*			
CAWG_04324	*MKK2*			
CAWG_00180	*SPA2*			
CAWG_01373	*MKC1*			
CAWG_03675	*MLP1*			
CAWG_03423	*PTP2*			
CAWG_02293	*PTP3*			
CAWG_00432	*CPP1*			

CAWG_03658	*RLM1*			
CAWG_05947	*PAF1*			
**CAWG_01201**	***SWI4***	**up**	**up**	**up**
CAWG_00558	*SWI6*			
CAWG_01443	*GSL2*			
CAWG_01142	*GSC1*			
CAWG_00845	*GSL1*			
